# Supplementary material for: Historical connections among river basins and climatic changes explain the biogeographic history of a water rat
Source: PeerJ. 2018 Jul 27;6:e5333. doi: 10.7717/peerj.5333 (PMC6065461; doi:10.7717/peerj.5333)
Supplement: Appendix S1 [file peerj-06-5333-s001.pdf]

PeerJ

SUPPORTING INFORMATION
Historical connections among river basins and climatic changes explain the biogeographic history of a water rat

APPENDIX S1: List of genetic samples of *Nectomys* used in the present study, organized alphabetically by species and localities. AB: Renata Pardini; CIT: Yatiyo Yassuda-Yonenaga; CTA: Coleção de Tecido Animal da Universidade Federal do Espírito Santo; ISSP: Israel de Souza Pinto; LBCE: Cibele R. Bonvicino; LGA, and CVMA: Valéria Fagundes; LPC: Leonora P. Costa; MBML: Museu de Biologia Professor Mello Leitão; MCNM: Museu de Ciências Naturais PUC-Minas; MP: Marcelo Passamani; MV: unknown; RM: Raquel Moura; ROD: Raisa Rodarte; TAX, and FER: Mariana Ferreira Rocha; YL: Yuri L. R. Leite. All samples designated with collector numbers are deposited in CTA collection.

Species - COUNTRY: STATE/DEPARTMENT: **Municipality (corresponding point number in Fig.1):** Specific Locality

Nectomys apicalis: NO INFORMATION: AY041195 (Genbank). PERU: CUSCO: **La Convención** (-72,83; -12,33): EU340013.

Nectomys rattus: PERU: LORETO: **Maynas** (-73,23; -4,30): MV 970012. BRAZIL: MATO GROSSO: **Aripuanã** (-59,3; -9,98): CIT 669. **Barra do Garças** (-52,35; -15,63): CTA 1348, 1366, 1381 and 1440. PIAUÍ: **Cristino Castro** (-44,16; -8,83): CIT 1386 and 1452. RONDÔNIA: **Porto Velho** (-63,9; -8,76): UFRO 583.

Nectomys squamipes: BRAZIL: BAHIA: **Cairu (1)**, Fazenda Subauma (-39,05; -13,50): RM 167. **Itamaraju (6)**, Fazenda Princesa do Pajaú (-39,53; -17,06): RM 212. **Itamari (3)**, Fazenda São Roque (-39,61; -13,78): RM 202. **Jussari (4)**, Serra do Teimoso (-39,48; -15,17): CIT 1574, 1586 and 1604. **Nova Viçosa (7)**, Fazenda Elma (-39,56; -17,97): CTA 74 to 81, 90, 91, 93, 756 and 757. Fazenda João Guarda (-39,57; -17,98): CTA 82. Fazenda Suécia (-40,03; -17,88) CTA 83, 84 and 89. **Una (5)**: ESMAI - Estação Experimental Lemos Maia (-39,08; -15,28): CTA 968. Reserva Particular do Patrimônio Natural Santa Angélica (-39,07; -15,29): YL 784, 788, 792, 793, 794, 806 and 833. **Wenceslau Guimarães (2)**: Estação Ecológica Estadual Nova Esperança (-

39,48; -13,68): RM 158. ESPÍRITO SANTO: **Águia Branca (23)**: Fazenda Lacerda, Águas Claras (-40,75; -18,98): CTA 128, 238 and 239. Fazenda do Zequinha Manduca (-40,81; -18,87): CTA 135 and 240. **Anchieta (31)**: Ubu, Samarco (-40,64; -20,80): MBML 2306. **Aracruz (26)**: Barragem da Fibria (-40,27; -19,82): LPC 1347. **Cariacica (29)**: Reserva Biológica de Duas Bocas (-40,52; -20,28): CTA 388, 508 to 518. **Castelo (32)**: Parque Estadual do Forno Grande (-41.18; -20.60): MBML 2685. **Conceição da Barra (8)**: Floresta Nacional do Rio Preto (-39,84; -18,35): LPC 1278, 1283, 1289, 1291, 1295, 1297 and 1300. **Ibitirama (33)**: Parque Nacional do Caparaó (-41,67; -20,54): LGA 1215, 1238 and 1239. **Linhares (25)**: Reserva da Vale (-40,07; -19,39): TAX 07, 17, 18 and 26. **Pancas (24):** Córrego São Luís (-40,84; -19,17): ISPP 1, 2, 3, 5, 8 and 9. **Pinheiros (10)**: Reserva Biológica Córrego do Veado (-40,22; -18,42): CVMA 7. **Presidente Kennedy (35)**: Alagados do Itabapoana (-41,03; -21,09): FER 01. **Santa Teresa (27)**: Estação Biológica de Santa Lúcia (-40,54; -19,96): CTA 803, LGA 60, 85, 95, 96, 140, 151. Valsugana Velha (-40,60; -19,93) MBML 2467, 2468 and 2685. **São José do Calçado (34)**: Sítio Sapucaia, Alegoria (-41,72; -21,04): LPC 1367 and 1375. Airituba (-41,67; -20,96): LPC 1391 and 1392. **São Mateus (9)** (-39,85; -18,72): LGA 2521. **Serra (28):** Clube Capixaba de Golfe, Área de Proteção Ambiental do Mestre Álvaro (-40,18; -20,07): YL 774. **Viana (30)**: Pimenta (-40,47; -20,38): CTA 616, 620, 719 to 727. Sítio Bem-te-vi, Formate (-40,50; -20,35): CTA 729 to 732. Fazenda Boa Baixa (-40,46; -20,39): CTA 733 to 735 and 858. Coacas (-40,47; -20,36): CTA 736 and 737. MATO GROSSO DO SUL: **Maracaju (49)**: Fazenda da Mata (-55,17; -21,61): AF181283 (Genbank). MINAS GERAIS: **Aiuruoca (45)** (-44,60; -21,92): MP 318. **Barão dos Cocais (17)**: Mina de Gongo Soco (-43,49; -19,95): MCNM 1425. **Barbacena (46)** (-43,77; -21,23): MCNM 2458. **Catas Altas (18)**: Área Alegria 2 (-43,40; -20,08) MCNM 1593. **Felixlândia (12)** (-44,88; -18,73) MCNM 2005. **Lagoa Santa (14)** (-43,89; -19,63) ML 67 and 83. **Marliéria (20):** Parque Estadual do Rio Doce (-42,65; -19,72): CTA 1119. **Nova Lima (15)** (-43,84; -20,01) MCNM 1920, 1928 and 1930. **Ouro Preto (19)**: Floresta Estadual do Uaimii (-43,48; -20,41): MCNM 1371. **Santa Bárbara (16)**: Parque do Caraça (-43,50; -20,08): CTA 925 and RM 02. **Santa Cruz do Escalvado (21)**: UHE Cadonga (-42,86; -20,22): MCNM 1362. **Santana do Riacho (13)**: Parque Nacional da Serra do Cipó (-43,71; -19,17): MCNM 2045. **Santo Antônio do Amparo (47):** (-44,92; -20,95): MP 331. **São Gonçalo do Rio Preto (11)**: Parque Estadual do Rio Preto (-43,38; -18,15): CTA 928, 930 and 1108. **Teixeiras (22)** (-42,86; -20,65): MCNM 303 and 304. RIO DE JANEIRO: **Sumidouro (36)** (-42,67; -22,04): LBCE 7781 and 7805. SÃO PAULO: **Cananéia (42)**: Parque Estadual Ilha do Cardoso (-47,96; -25,13): ROD 131, 135, 150, 154, 297 and GU126522 (Genbank). **Ilhabela (39)**: Fazenda da Toca, Ilha de São Sebastião (-45,32; -23,84): ML 47.

Juquiá (41) (-47,63; -24,31): CIT 1302 (donated sequence). **Luiz Antônio (48)** (-47,72; -21,55): CIT 937 (donated sequence). **São Luís do Paraitinga (37)**: Parque Estadual da Serra do Mar (-45,12; -23,36): ROD 41. **Sete Barras (40):** Fazenda Intervales (-48,08; -24,22): CIT 02, 41 and 100 (donated sequences). **Sorocaba (44):** Floresta Nacional de Ipanema (-47,63; -23,43) CTA 1737 to 1739, 1745 to 1747, 1751, 1757 and LPC 857. **Tapiraí (43):** Sítio D. Ivone (-47,45; -23,85): AB 598. **Ubatuba (38)**: Praia do Félix (-45,08; -23,43): ML 61. PARAGUAY: PARAGUARI: **Ybycuí (50)**: Parque Nacional de Ybycuí (-56,86; -26,07) EU074634 (Genbank).
