# Supplementary material for: Historical connections among river basins and climatic changes explain the biogeographic history of a water rat
Source: PeerJ. 2018 Jul 27;6:e5333. doi: 10.7717/peerj.5333 (PMC6065461; doi:10.7717/peerj.5333)
Supplement: Appendix S2 [file peerj-06-5333-s002.pdf]

PeerJ

SUPPORTING INFORMATION
Historical connections among river basins and climatic changes explain the biogeographic history of a water rat

APPENDIX S2: Amplification profiles for the genetic markers (Table S2.1); localities used in species distribution modelling (Table S2.2); Genbank accession number for the sequences used (Table S2.3); samples and size alleles for all microsatellite loci (Table S2.4); deviations from Hardy-Weinberg equilibrium and null alleles frequency for the six microsatellite loci (Table S2.5); basic descriptive statistics for the six microsatellite loci (Table S2.6); genetic distances (p-distance) for mtDNA (Table S2.7); Dates of major cladogenesis for Cyt b related to *Nectomys* (Table S2.8); neutrality tests (Table S2.9); heterozygote rates for microsatellite data (Table S2.10); F_{ST} and R_{ST} values among populations of *Nectomys squamipes* from different basins in the Atlantic Forest (Table S2.11); validation indices of the SDM (Table S2.12).

Table S2.1: Amplification profiles for the mitochondrial genes cytochrome b (Cyt b) and D-loop and for the six microsatellite loci from *Nectomys squamipes* (Nec). DNA was extracted using the protocol described by Bruford *et al.* (1992). For Cyt b we used the primers MVZ05 and MVZ16 (Smith & Patton, 1993) and for D-loop the primers L0 (Douzery & Randi, 1997) and E3 (Huchon *et al.*, 1999). Primers for Nec12, Nec14, Nec15, Nec18 were designed by Almeida *et al.* (2000), and Nec19 and Nec23 by Maroja *et al.* (2003).

Marker	Initial denaturation	Cycles	Denaturation	Annealing	Extension	Final extension
**Cyt b**	94 °C - 5min	40	94 °C - 30s	48 °C - 30s	72 °C - 45s	72 °C - 5min
**D-loop**	94 °C - 3min	36	94 °C - 30s	52 °C - 30s	72 °C - 30s	72 °C - 3min
**Nec12**	94 °C - 5min	30	94 °C - 15s	61 °C - 30s	72 °C - 30s	72 °C - 4min
**Nec14**	94 °C - 5min	30	94 °C - 15s	61 °C - 30s	72 °C - 30s	72 °C - 4min
**Nec15**	94 °C - 5min	34	94 °C - 15s	58 °C - 30s	72 °C - 30s	72 °C - 4min
**Nec18**	94 °C - 5min	35	94 °C - 15s	58 °C - 30s	72 °C - 30s	72 °C - 4min
**Nec19**	94 °C - 5min	31	94 °C - 30s	58 °C - 30s	72 °C - 30s	72 °C - 4min
**Nec23***	94 °C - 5min	7	94 °C - 60s	55 °C - 60s	72 °C - 60s	72 °C - 4min
		30	94 °C - 60s	57 °C - 60s	72 °C - 60s	

*Amplification performed in two steps for Nec23.

REFERENCES:

Almeida F.C., Maroja L.S., Seuánez H.N., Cerqueira R., & Moreira M.A.M. (2000) Identification of microsatellite loci in the water-rat *Nectomys squamipes* (Rodentia, Sigmodontinae). *Molecular Ecology*, **9**, 2172–2173.

Bruford M.W., Hanotte O., Brookfield J.F.Y., & Burke T. (1992) Single-locus and multilocus DNA fingerprinting. *Molecular genetics analyses of populations* (ed. by A.R. Hoelzel), pp. 225–269. IRL Press, Oxford.

Douzery E. & Randi E. (1997) The mitochondrial control region of Cervidae: evolutionary patterns and phylogenetic content. *Molecular Biology and Evolution*, **14**, 1154–1166.

Huchon D., Delsuc F., Catzeflis F.M., & Douzery E.J.P. (1999) Armadillos exhibit less genetic polymorphism in North America than in South America: Nuclear and mitochondrial data confirm a founder effect in *Dasypus novemcinctus* (Xenarthra). *Molecular Ecology*, **8**, 1743–1748.

Maroja L.S., Almeida F.C., Cerqueira R., Seuánez H.N., & Moreira M.A.M. (2003) *Nectomys squamipes* microsatellites and homologous loci in sigmodontine rodents. *Journal of Heredity*, **94**, 171–174.

Smith M.F. & Patton J.L. (1993) The diversification of South American murid rodents: evidence from mitochondrial DNA sequence data for the akodontine tribe. *Biological Journal of the Linnean Society*, **50**, 149–177.

Table S2.2: List of the 109 localities used in species distribution modelling of *Nectomys squamipes*, organized alphabetically by country, state/province/department and municipalities. Localities in bold (40) were used for external validation and the other 69 localities were used in MaxEnt. APN-AR: Administración de Parques Nacionales, Argentina. MAMÍFEROS – ES: Espírito Santo mammals database. MBML: Museu de Biologia Professor Mello Leitão, Brazil. MVZ: Museum of Vertebrate Zoology (University of California, Berkeley), USA. MZUEL: Museu de Zoologia, Universidade Estadual de Londrina, Brazil. MCNM: Museu de Ciências Naturais, PUC-Minas, Brazil. SINBIOTA: Biota-Fapesp Information System, Brazil. UFES-CTA: animal tissue collection, Universidade Federal do Espírito Santo, Brazil. UFES-MAM: mammal collection, Universidade Federal do Espírito Santo, Brazil. UFMG: Universidade Federal de Minas Gerais, Brazil. ZUEC-MAM: Museu de Zoologia Professor Adão José Cardoso, Universidade Estadual de Campinas, Brazil.

Long	Lat	Country	State/Province	Municipality	Source
-54.95250	-27.08800	Argentina	Misiones	Cainguás	Cirignoli *et al.* 2011
-54.63333	-25.66667	Argentina	Misiones	Iguazú	Robles and Navone 2014
-54.21667	-26.93333	Argentina	Misiones	Guaraní	Robles and Navone 2014
-53.91667	-27.13333	Argentina	Misiones	Guaraní	Robles and Navone 2014
-53.40639	-26.74778	Brazil	Paraná	São Miguel do Oeste	Spezia *et al.* 2013
-53.30316	-27.94148	Brazil	Rio Grande do Sul	Palmeiras das Missões	Kionka 2013
-53.02959	-27.99657	Brazil	Rio Grande do Sul	Nova Boa Vista	Peters *et al.* 2010
-52.70000	-27.13333	Brazil	Santa Catarina	Chapecó	Maestri *et al.* 2015
-52.22444	-28.04194	Brazil	Rio Grande do Sul	Sertão	Luza *et al.* 2013
-49.92639	-24.57278	Brazil	Paraná	Piraí do Sul	Grazzini *et al.* 2015
-49.52444	-26.47389	Brazil	Santa Catarina	Rio Negrinho	Nicola 2009
-49.21841	-27.02243	Brazil	Santa Catarina	Indaial	Barbosa 2012
-49.14278	-24.71306	Brazil	Paraná	Adrianópolis	Onofrio *et al.* 2013
-48.91667	-25.43333	Brazil	Paraná	Morretes	Cerboncini 2012
-48.80000	-27.73333	Brazil	Santa Catarina	Santo Amaro da Imperatriz	Kuhnen *et al.* 2012
-48.63944	-25.61444	Brazil	Paraná	Paranaguá	Mochi-Junior 2014
-47.97556	-24.13370	Brazil	São Paulo	Sete Barras	Vieira 1999
-47.96667	-25.13333	Brazil	São Paulo	Cananéia	UFES-MAM
-47.62806	-23.43528	Brazil	São Paulo	Iperó	MVZ
-47.61667	-21.61667	Brazil	São Paulo	Santa Rita do Passa Quatro	Lyra Jorge *et al.* 2001
-47.09250	-23.71490	Brazil	São Paulo	Ibiúna	SINBIOTA
-46.96764	-23.74666	Brazil	São Paulo	Cotia	Umetsú *et al.* 2006
-45.90000	-23.65000	Brazil	São Paulo	Salesópolis	MVZ

Long	Lat	Country	State/Province	Municipality	Source
-45.12500	-23.35833	Brazil	São Paulo	São Luís do Paraitinga	UFES-MAM
-44.83386	-20.89684	Brazil	Minas Gerais	Santo Antônio do Amparo	Rocha *et al.* 2011
-44.83190	-23.36600	Brazil	São Paulo	Ubatuba	Pinheiro and Geise 2008
-44.77957	-18.87376	Brazil	Minas Gerais	Felixlândia	MCNM
-44.64641	-22.08745	Brazil	Minas Gerais	Aiuruoca	MCNM
-44.56159	-21.59679	Brazil	Minas Gerais	Minduri	Machado *et al.* 2013
-44.37810	-22.88700	Brazil	Rio de Janeiro	Angra dos Reis	Cunha and Rajão 2007
-43.77465	-22.68160	Brazil	Rio de Janeiro	Paracambi	MCNM
-43.50000	-20.08333	Brazil	Minas Gerais	Santa Bárbara	UFES-CTA
-43.38333	-18.15000	Brazil	Minas Gerais	São Gonçalo do Rio Preto	UFES-CTA
-42.68115	-22.02763	Brazil	Rio de Janeiro	Sumidouro	Bonecker *et al.* 2009
-42.65000	-19.71667	Brazil	Minas Gerais	Mariléia	MVZ
-42.49801	-22.49807	Brazil	Rio de Janeiro	Silva Jardim	Galliez *et al.* 2009
-42.30611	-22.53666	Brazil	Rio de Janeiro	Silva Jardim	Pessôa *et al.* 2010
-42.02667	-22.43750	Brazil	Rio de Janeiro	Casemiro de Abreu	Pessôa *et al.* 2010
-42.01694	-22.53750	Brazil	Rio de Janeiro	Casemiro de Abreu	Pessôa *et al.* 2010
-42.00550	-22.30250	Brazil	Rio de Janeiro	Macaé	Pessôa *et al.* 2010
-41.72104	-22.28760	Brazil	Rio de Janeiro	Macaé	Pessôa *et al.* 2010
-41.71917	-21.04250	Brazil	Espírito Santo	São José do Calçado	UFES-MAM
-41.70306	-20.49722	Brazil	Espírito Santo	Ibitirama	UFES-MAM
-41.67083	-20.95806	Brazil	Espírito Santo	São José do Calçado	UFES-MAM
-41.44055	-21.79160	Brazil	Rio de Janeiro	Campos do Goytacazes	Pessôa *et al.* 2010
-41.40300	-22.07020	Brazil	Rio de Janeiro	Quissamã	Pessôa *et al.* 2010
-41.39770	-12.54750	Brazil	Bahia	Lençóis	Pereira 2006
-41.24910	-16.71800	Brazil	Bahia	Joaíma	Pereira 2006
-41.18470	-20.60360	Brazil	Espírito Santo	Castelo	UFES-MAM
-40.84588	-19.16943	Brazil	Espírito Santo	Pancas	UFES-MAM
-40.78611	-18.87472	Brazil	Espírito Santo	Águia Branca	UFES-MAM
-40.71972	-18.97972	Brazil	Espírito Santo	Águia Branca	UFES-MAM
-40.59528	-20.77361	Brazil	Espírito Santo	Anchieta	MAMÍFEROS-ES
-40.57053	-19.96522	Brazil	Espírito Santo	Santa Teresa	MAMÍFEROS-ES
-40.51139	-20.28111	Brazil	Espírito Santo	Cariacica	UFES-MAM
-40.46500	-20.37917	Brazil	Espírito Santo	Viana	UFES-MAM
-40.30780	-20.12860	Brazil	Espírito Santo	Serra	UFES-MAM
-40.23333	-20.23333	Brazil	Espírito Santo	Serra	UFES-MAM
-40.01000	-19.09660	Brazil	Espírito Santo	Linhares	MAMÍFEROS-ES
-40.00694	-17.87889	Brazil	Bahia	Nova Viçosa	UFES-MAM
-39.84460	-18.35517	Brazil	Espírito Santo	Conceição da Barra	UFES-MAM
-39.78613	-19.55822	Brazil	Espírito Santo	Linhares	UFES-MAM
-39.71667	-13.60000	Brazil	Bahia	Wenceslau Guimarães	UFMG
-39.66667	-13.85000	Brazil	Bahia	Itamari	UFMG
-39.56611	-17.97889	Brazil	Bahia	Nova Viçosa	UFES-MAM
-39.38333	-15.23333	Brazil	Bahia	Una	UFMG

Long	Lat	Country	State/Province	Municipality	Source
-39.16583	-15.20639	Brazil	Bahia	Una	UFMG
-39.08333	-15.28333	Brazil	Bahia	Una	UFES-CTA
-39.03333	-13.51667	Brazil	Bahia	Cairu	UFMG
**-54.45024**	**-21.44955**	**Brazil**	**Mato Grosso do Sul**	**Nova Alvorada do Sul**	**Cárceres *et al.* 2008**
**-54.44021**	**-25.68314**	**Argentina**	**Misiones**	**Iguazú**	**APN-AR**
**-54.16889**	**-26.93833**	**Argentina**	**Misiones**	**Guaraní**	**APN-AR**
**-52.89250**	**-18.26389**	**Brazil**	**Goiás**	**Mineiros**	**Rodrigues *et al*. 2002**
**-52.78191**	**-17.63404**	**Brazil**	**Goiás**	**Mineiros**	**Cárceres *et al.* 2008**
**-51.25000**	**-23.45000**	**Brazil**	**Paraná**	**Londrina**	**MZUEL**
**-48.65000**	**-26.15000**	**Brazil**	**Santa Catarina**	**Itapoá**	**Graipel *et al.* 2014**
**-48.21420**	**-24.22530**	**Brazil**	**São Paulo**	**Sete Barras**	**ZUEC-MAM**
**-47.92670**	**-15.73140**	**Brazil**	**Distrito Federal**	**Brasília**	**Barbosa 2012**
**-47.32138**	**-16.20778**	**Brazil**	**Minas Gerais**	**Cabeceira Grande**	**MCNM**
**-45.90000**	**-15.38333**	**Brazil**	**Minas Gerais**	**Formoso**	**Carmignotto 2004**
**-44.82887**	**-23.36328**	**Brazil**	**São Paulo**	**Ubatuba**	**Penheiro and Geise 2008**
**-44.63330**	**-22.35920**	**Brazil**	**Rio de Janeiro**	**Itatiaia**	**Geise *et al.* 2004**
**-44.56670**	**-22.25000**	**Brazil**	**Minas Gerais**	**Bocaina de Minas**	**Geise *et al.* 2004**
**-44.03860**	**-20.10200**	**Brazil**	**Minas Gerais**	**Brumadinho**	**MCNM**
**-43.95000**	**-20.25000**	**Brazil**	**Minas Gerais**	**Itabirito**	**MCNM**
**-43.87861**	**-20.15417**	**Brazil**	**Minas Gerais**	**Nova Lima**	**MCNM**
**-43.63080**	**-19.34250**	**Brazil**	**Minas Gerais**	**Santana do Riacho**	**MCNM**
**-43.59889**	**-19.96361**	**Brazil**	**Minas Gerais**	**Barão de Cocais**	**MCNM**
**-43.58690**	**-20.24860**	**Brazil**	**Minas Gerais**	**Ouro Preto**	**MCNM**
**-43.38417**	**-19.87333**	**Brazil**	**Minas Gerais**	**São Gonçalo do Rio Abaixo**	**MCNM**
**-43.23333**	**-19.94833**	**Brazil**	**Minas Gerais**	**Rio Piracicaba**	**MCNM**
**-42.98750**	**-22.44890**	**Brazil**	**Rio de Janeiro**	**Teresópolis**	**Vaz *et al.* 2007**
**-42.87535**	**-20.21903**	**Brazil**	**Minas Gerais**	**Santa Cruz da Escalvado**	**MCNM**
**-42.87330**	**-22.28890**	**Brazil**	**Rio de Janeiro**	**Teresópolis**	**Vaz *et al.* 2007**
**-42.85192**	**-19.08539**	**Brazil**	**Minas Gerais**	**Dores de Guanhães**	**MCNM**
**-42.68716**	**-19.09878**	**Brazil**	**Minas Gerais**	**Braúnas**	**MCNM**
**-42.67510**	**-16.77525**	**Brazil**	**Minas Gerais**	**Cristália**	**MCNM**
**-41.87000**	**-21.93000**	**Brazil**	**Rio de Janeiro**	**Santa Maria Madalena**	**Modesto *et al.* 2008**
**-41.64333**	**-22.26694**	**Brazil**	**Rio de Janeiro**	**Carapebus**	**Pessôa *et al.* 2010**
**-41.18572**	**-19.50859**	**Brazil**	**Minas Gerais**	**Aimorés**	**MCNM**
**-40.85556**	**-16.05972**	**Brazil**	**Minas Gerais**	**Almenara**	**MCNM**
**-40.53170**	**-20.11440**	**Brazil**	**Espírito Santo**	**Santa Leopoldina**	**MBML**
**-40.42140**	**-20.60000**	**Brazil**	**Espírito Santo**	**Guarapari**	**Venturini *et al.* 1996**
**-40.38165**	**-19.88372**	**Brazil**	**Espírito Santo**	**Ibiraçu**	**MBML**
**-40.28727**	**-17.83954**	**Brazil**	**Minas Gerais**	**Nanuque**	**MCNM**
**-40.07242**	**-19.84014**	**Brazil**	**Espírito Santo**	**Aracruz**	**UFES-MAM**
**-40.06180**	**-16.39980**	**Brazil**	**Minas Gerais**	**Santa Maria do Salto**	**MCNM**
**-40.06143**	**-19.14870**	**Brazil**	**Espírito Santo**	**Linhares**	**UFES-MAM**
**-37.33560**	**-10.75220**	**Brazil**	**Sergipe**	**Areia Branca**	**Oliveira *et al.* 2005**

REFERENCES

Barbosa LM (2012) *Filogenia e identificação de roedores Sigmodontinae através de marcadores moleculares*: *avaliação do código de barras de DNA*. Master's Thesis, Universidade Federal do Rio Grande do Sul, Porto Alegre.

Bonecker ST, Portugal LG, Costa-Neto SF, Gentile R (2009) A long term study of small mammal populations in a Brazilian agricultural landscape. *Mammalian Biology*, **74**, 467–477.

Cáceres NC, Casella J, Vargas CF, Prates LZ, Tombini AA, Goulart CS, Lopes WH (2008). Distribuição geográfica de pequenos mamíferos não voadores nas bacias dos rios Araguaia e Paraná, região centro-sul do Brasil. *Iheringia, Série Zoologia*, **98**, 173-180.

Carmignotto AP (2004) *Pequenos mamíferos terrestres do bioma Cerrado*: *padrões faunísticos locais e regionais*. PhD Thesis, Universidade de São Paulo, São Paulo.

Cerboncini RAS (2013) Respostas de pequenos mamíferos ao efeito de borda da ferrovia Paranaguá-Curitiba no Parque Estadual Pico do Marumbi, Morretes-PR. Master's Thesis. Universidade Federal do Paraná, Curitiba.

Cirignoli S, Galliari CA, Pardiñas UFJ, Podestá DH, Abramson R (2011) Mamíferos de la Reserva Valle del Cuña Pirú, Misiones, Argentina. *Mastozoologia Neotropical*, **18**, 25-43.

Cunha AA, Rajão H (2007) Mamíferos terrestres e aves da Terra Indígena Sapukai (Aldeia Guarani do Bracui), Angra dos Reis, RJ, Brasil. *Boletim do Museu de Biologia Mello Leitão (Nova Série)*, **21**, 19-34.

Galliez M, Leite MS, Queiroz TL, Fernandez FAS (2009) Ecology of the water opossum *Chironectes minimus* in Atlantic forest streams of southeastern Brazil. *Journal of Mammalogy*, **90**, 93-103.

Geise L, Pereira LG, Bossi DPE, Bergallo HG (2004) Pattern of elevational distribution and richness of non volant mammals in Itatiaia National Park and its surroundings, in southeastern Brazil. *Brazilian Journal of Biology*, **64**, 599-612.

Graipel ME, Hernández MIM, Salvador C (2014) Evaluation of abundance indexes in open population studies: a comparison in populations of small mammals in southern Brazil. *Brazilian Journal of Biology*, **74**(3), 553-559.

Grazzini G, Mochi-Junior CM, Oliveira HD, Pontes JDS, Gatto-Almeida F, Tiepolo LM (2015) Identidade, riqueza e abundãncia de pequenos mamíferos (Rodentia e Didelphimorphia) de área de Floresta com Araucária no estado do Paraná, Brasil. *Papéis Avulsos de Zoologia (São Paulo)*, **55**(15), 217-230.

Kionka DCO (2013) *Mastofauna não-voadora em fragmentos florestais nativos circundados por uma matriz agrícola e de pastagem em Palmeira das Missões/RS*. Master's Thesis. Centro Universitário UNIVATES, Lajeado.

Kuhnen VV, Graipel ME, Pinto CJC (2012) Differences in richness and composition of gastrointestinal parasites of small rodents (Cricetidae, Rodentia) in a continental and insular area of the Atlantic Forest in Santa Catarina state, Brazil. *Brazilian Journal of Biology*, **72**(3), 563-567.

Luza AL, Zanella N, Christoff AU, Barreto-Lima AF, Grando JV (2013) Relação entre fatores exógenos e a abundância de roedores em remanescente de Floresta Ombrófila Mista, Rio Grande do Sul. *Revista Brasileira de Biociências*, **11**(3).

Lyra Jorge MC, Pivello VR, Meirelles ST, Vivo M (2001) Riqueza e abundância de pequenos mamíferos em ambientes de cerrado e floresta, na Reserva Cerrado Pé-de-gigante, Parque Estadual de Vassununga (Santa Rita do Passa Quatro, SP). *Naturalia*, **26**, 287-302.

Machado FS, Gregorin R, Mouallem PSB (2013) Small mammals in high altitude phytophysiognomies in southeastern Brazil: are heterogeneous habitats more diverse? *Biodiversity and Conservation*, **22**(8), 1769-1782.

Maestri R, Galiano D, Kubiak BB, Marinho JR (2014) Diversity of small land mammals in a subtropical Atlantic forest in the western region of the state of Santa Catarina, southern Brazil. *Biota Neotropica*, **14**(4), 1-7.

Mochi-Junior CM (2014) *Composição taxonômica e avaliação da diversidade da fauna de pequenos mamíferos não-voadores na formação submontana do Parque Nacional Saint-Hilaire/Lange, Mata Atlântica costeira do Paraná*. Master's Thesis. Universidade Federal do Paraná, Curitiba.

Modesto TC, Pessôa FS, EnriciII MC, Attias N, Jordão-Nogueira T, Costa LM, Albuquerque HG, Bergallo HG (2008) Mamíferos do Parque Estadual do Desengano, Rio de Janeiro, Brasil. *Biota Neotropica*, **8**.

Nicola PA (2009) *Comunidades de pequenos mamíferos como indicadores de qualidade ambiental no planalto norte catarinense*. PhD Thesis. Universidade Federal do Paraná, Curitiba.

Oliveira FF, Ferrari SF, da Silva SDB (2005) Mamíferos não-voadores. In: Carvalho CD and Vilar JC (coord.) *Parque Nacional Serra de Itabaiana–Levantamento da Biota*. Aracaju: IBAMA. pp. 77-91.

Onofrio VC, Nieri-Bastos FA, Sampaio JDS, Soares JF, Silva MJDJ, Barros-Battesti DM (2013) Noteworthy records of *Ixodes schulzei* (Acari: Ixodidae) on rodents from the State of Parana, southern Brazil. *Revista Brasileira de Parasitologia Veterinária*, **22**(1), 159-161.

Pereira LG (2006) *Chapada Diamantina e Vale do Rio Jequitinhonha: Composição da mastofauna e estrutura microevolutiva de algumas populações de pequenos mamíferos*. PhD Thesis, Universidade Federal do Rio de Janeiro, Rio de Janeiro.

Pessôa LM, Tavares WC, Gonçalves PR (2010) Mamíferos das restingas do macrocompartimento litorâneo da bacia de Campos, Rio de Janeiro. In: *Mamíferos de restingas e manguezais do Brasil* (Eds. Pessôa LM, Tavares WC & Siciliano S.), p. 95-125. Sociedade Brasileira de Mastozoologia, Rio de Janeiro.

Peters FB, Roth PRO, Machado LF, Coelho EL, Jung DMH, Christoff AU (2010) Assembléia de mamíferos dos agroecossistemas constituintes da bacia hidrográfica do rio da Várzea, Rio Grande do Sul. *Biotemas*, **23**, 91-107.

Pinheiro PS, Geise L (2008) Non-volant mammals of Picinguaba, Ubatuba, state of São Paulo, southeastern Brazil. *Boletim do Museu de Biologia Mello Leitão (Nova Série)*, **23**, 51-59.

Robles MDR, Navone GT (2014). New host records and geographic distribution of species of *Trichuris* (Nematoda: Trichuriidae) in rodents from Argentina with an updated summary of records from America. *Mastozoología Neotropical*, **21**(1), 67-78.

Rocha MF, Passamani M, Louzada J (2011) A Small Mammal Community in a Forest Fragment, Vegetation Corridor and Coffee Matrix System in the Brazilian Atlantic Forest. *PLoS One*, **6**, e23312.

Rodrigues FHG, Silveira L, Jácomo ATA, Carmignotto AP, Bezerra AMR, *et al.* (2002). Composição e caracterização da fauna de mamíferos do Parque Nacional das Emas, Goiás, Brasil. *Revista Brasileira de Zoologia*, **19**(2), 589-600.

Spezia MB, Grasel D, Miranda G (2013) Inventário rápido de mamíferos não voadores em um fragmento florestal do bioma Mata Atlântica. *Unoesc & Ciência-ACBS*, **4**(2), 145-154.

Umetsú F, Naxara L, Pardini R (2006) Evaluating the efficiency of pitfall traps for sampling small mammals in the neotropics. *Journal of Mammalogy*, **87**, 757-765.

Vaz VC, D'Andrea PS, Jansen AM (2007) Effects of habitat fragmentation on wild mammal infection by *Trypanosoma cruzi*. *Parasitology*, **134**, 1785-1793.

Venturini AC, Ofranti AMS, Varejão JBM, Paz PR (1996) *Aves e mamíferos na restinga: Parque Estadual Paulo César Vinha – Setiba, Guarapari, ES*. Secretaria de Estado de Desenvolvimento Sustentável, Vitória.

Vieira EM (1999) *Estudo comparativo de comunidades de pequenos mamíferos em duas áreas de Mata Atlântica situadas a diferentes altitudes no Sudeste do Brasil*. PhD Thesis, Universidade Estadual de Campinas, Campinas.

Table S2.3: Genbank accession number for the sequences of Cytb and D-loop used in this study. Sequences in bold were produced, and the haplotype list of Cytb is the same used in the Figure 4.

Species	Genbank Accession Number	Marker	Sample ID	Haplotypes
Nectomys squamipes	**KY498357**	**Cyt b**	**AB598**	**H01**
Nectomys squamipes	**KY498358**	**Cyt b**	**CIT1574 and CIT1586**	**H02**
Nectomys squamipes	**KY498359**	**Cyt b**	**CIT1604**	**H03**
Nectomys squamipes	**KY498360**	**Cyt b**	**CTA75; CTA76; CTA79; CTA81; CTA89; CTA93; CTA756; CTA757; LPC1289 and LPC1300**	**H04**
Nectomys squamipes	**KY498361**	**Cyt b**	**CTA77**	**H05**
Nectomys squamipes	**KY498362**	**Cyt b**	**CTA91**	**H06**
Nectomys squamipes	**KY498363**	**Cyt b**	**CTA128 and CTA239**	**H07**
Nectomys squamipes	**KY498364**	**Cyt b**	**CTA509; CTA510; CTA512; CTA514; CTA517; CTA616; CTA719; CTA728; CTA730; CTA731; CTA732; CTA733; CTA735; CTA858 and YL774**	**H08**
Nectomys squamipes	**KY498365**	**Cyt b**	**CTA968; LGA85; LGA2521; MCNM1425; MP318 and YL784**	**H09**
Nectomys squamipes	**KY498366**	**Cyt b**	**CTA1119**	**H10**
Nectomys squamipes	**KY498367**	**Cyt b**	**CTA1737; CTA1739; CTA1746; CTA1747 and CTA1751**	**H11**
Nectomys squamipes	**KY498368**	**Cyt b**	**ISSP05**	**H12**
Nectomys squamipes	**KY498369**	**Cyt b**	**LBCE7805**	**H13**
Nectomys squamipes	**KY498370**	**Cyt b**	**LGA60; LGA95 and LGA96**	**H14**
Nectomys squamipes	**KY498371**	**Cyt b**	**LGA1215 and LGA1239**	**H15**
Nectomys squamipes	**KY498372**	**Cyt b**	**LGA1238**	**H16**
Nectomys squamipes	**KY498373**	**Cyt b**	**LPC1392**	**H17**
Nectomys squamipes	**KY498374**	**Cyt b**	**MBML2306**	**H18**
Nectomys squamipes	**KY498375**	**Cyt b**	**MBML2685**	**H19**
Nectomys squamipes	**KY498376**	**Cyt b**	**MCNM303 and MCNM304**	**H20**
Nectomys squamipes	**KY498377**	**Cyt b**	**MCNM1371**	**H21**
Nectomys squamipes	**KY498379**	**Cyt b**	**MCNM1920 and MCNM1930**	**H22**
Nectomys squamipes	**KY498380**	**Cyt b**	**MCNM2005**	**H23**
Nectomys squamipes	**KY498381**	**Cyt b**	**MCNM2045**	**H24**
Nectomys squamipes	**KY498382**	**Cyt b**	**ML61**	**H25**
Nectomys squamipes	**KY498383**	**Cyt b**	**RM167**	**H26**
Nectomys squamipes	**KY498384**	**Cyt b**	**RM202**	**H27**
Nectomys squamipes	**KY498385**	**Cyt b**	**RM212**	**H28**
Nectomys squamipes	EU340012	Cyt b	ROD154; ROD297 and FMNH141632	H29
Nectomys squamipes	**KY498386**	**Cyt b**	**TAX07**	**H30**
Nectomys squamipes	**KY498387**	**Cyt b**	**TAX17**	**H31**
Nectomys squamipes	**KY498388**	**Cyt b**	**TAX18**	**H32**
Nectomys squamipes	**KY498389**	**Cyt b**	**YL788**	**H33**
Nectomys squamipes	**KY498390**	**Cyt b**	**YL792**	**H34**
Nectomys squamipes	EU074634	Cyt b	TK63841	H35
Nectomys squamipes	AF181283	Cyt b	CRB540	H36
Nectomys squamipes	**KY498378**	**Cyt b**	**MCNM1593**	-
Nectomys squamipes	**KY498391**	**Cyt b**	**CIT02**	-
Nectomys squamipes	**KY498392**	**Cyt b**	**CIT41**	-
Nectomys squamipes	**KY498393**	**Cyt b**	**CIT100**	-

Species	Genbank Accession Number	Marker	Sample ID	Haplotypes
Nectomys squamipes	**KY498403**	**Cyt b**	**CIT937**	**-**
Nectomys squamipes	**KY498394**	**Cyt b**	**CIT1302**	**-**
Nectomys rattus	**KY498401**	**Cyt b**	**MV970012**	**-**
Nectomys rattus	**KY498395**	**Cyt b**	**CIT669**	**-**
Nectomys rattus	**KY498396**	**Cyt b**	**CIT1386**	**-**
Nectomys rattus	**KY498397**	**Cyt b**	**CIT1452**	**-**
Nectomys rattus	**KY498398**	**Cyt b**	**CTA1348**	**-**
Nectomys rattus	**KY498399**	**Cyt b**	**CTA1381**	**-**
Nectomys rattus	**KY498400**	**Cyt b**	**CTA1440**	**-**
Nectomys rattus	**KY498402**	**Cyt b**	**UFRO583**	**-**
Nectomys apicalis	EU340013	Cyt b	MVZ166700	-
Nectomys apicalis	AY041195	Cyt b	NK13407	-
Amphinectomys savamis	EU579480	Cyt b	MV97005	-
Cerradomys subflavus	AF181274	Cyt b	CEG42	-
Holochilus chacarius	GU185898	Cyt b	Roro007	-
Holochilus sciureus	EU579497	Cyt b	NK102248	-
Oligoryzomys chacoensis	GU185904	Cyt b	Or22498	-
Oligoryzomys flavescens	GU185924	Cyt b	UP51	-
Oligoryzomys nigripes	GU185910	Cyt b	LIF122	-
Pseudoryzomys simplex	EU579516	Cyt b	TK62425	-
Sooretamys angouya	EU579511	Cyt b	MNRJ50234	-
Nectomys squamipes	**KY498407**	**D-loop**	**AB598**	-
Nectomys squamipes	**KY498408**	**D-loop**	**CIT1574 and CIT1586**	-
Nectomys squamipes	**KY498409**	**D-loop**	**CIT1604**	-
Nectomys squamipes	**KY498410**	**D-loop**	**CTA74; CTA77; CTA80 and LPC1291**	-
Nectomys squamipes	**KY498411**	**D-loop**	**CTA75; CTA76; CTA79; CTA89; CTA90; CTA93; CTA756 and CTA757**	-
Nectomys squamipes	**KY498412**	**D-loop**	**CTA78; CTA81; LPC1289; LPC1295; LPC1297 and LPC1300**	-
Nectomys squamipes	**KY498413**	**D-loop**	**CTA82**	-
Nectomys squamipes	**KY498414**	**D-loop**	**CTA83**	-
Nectomys squamipes	**KY498415**	**D-loop**	**CTA84**	-
Nectomys squamipes	**KY498416**	**D-loop**	**CTA91**	-
Nectomys squamipes	**KY498417**	**D-loop**	**CTA128 and CTA239**	-
Nectomys squamipes	**KY498418**	**D-loop**	**CTA135 and CTA240**	-
Nectomys squamipes	**KY498419**	**D-loop**	**CTA238**	-
Nectomys squamipes	**KY498420**	**D-loop**	**CTA388**	-
Nectomys squamipes	**KY498421**	**D-loop**	**CTA508; CTA509; CTA510; CTA511; CTA512; CTA513; CTA514; CTA515; CTA516; CTA517; CTA518 and CTA729**	-
Nectomys squamipes	**KY498422**	**D-loop**	**CTA616 and MCNM2458**	-
Nectomys squamipes	**KY498423**	**D-loop**	**CTA620; CTA723; CTA726 and CTA858**	-
Nectomys squamipes	**KY498424**	**D-loop**	**CTA719; CTA720; CTA721; CTA722; CTA725; CTA727; CTA728; CTA730; CTA733; CTA734; CTA735; CTA736 and CTA737**	-
Nectomys squamipes	**KY498425**	**D-loop**	**CTA724**	-
Nectomys squamipes	**KY498426**	**D-loop**	**CTA731**	-
Nectomys squamipes	**KY498427**	**D-loop**	**CTA803; LGA60; LGA95; LGA96 and LGA151**	-
Nectomys squamipes	**KY498428**	**D-loop**	**CTA925**	-
Nectomys squamipes	**KY498429**	**D-loop**	**CTA928**	-

Species	Genbank Accession Number	Marker	Sample ID	Haplotypes
Nectomys squamipes	KY498430	D-loop	CTA930	-
Nectomys squamipes	KY498431	D-loop	CTA968 and YL784	-
Nectomys squamipes	KY498432	D-loop	CTA1108	-
Nectomys squamipes	KY498433	D-loop	CTA1119	-
Nectomys squamipes	KY498434	D-loop	CTA1737; CTA1738; CTA1739; CTA1746; CTA1747; CTA1751; and CTA1757	-
Nectomys squamipes	KY498435	D-loop	CTA1745; LPC857 and ROD41	-
Nectomys squamipes	KY498436	D-loop	CVMA07	-
Nectomys squamipes	KY498437	D-loop	FER01 and MCNM1425	-
Nectomys squamipes	KY498438	D-loop	ISSP01; ISSP02; ISSP03; ISSP05; ISSP08 and ISSP09	-
Nectomys squamipes	KY498439	D-loop	LBCE7781; LPC1391 and LPC1392	-
Nectomys squamipes	KY498440	D-loop	LGA85	-
Nectomys squamipes	KY498441	D-loop	LGA140 and MBML2468	-
Nectomys squamipes	KY498442	D-loop	LGA1215; LGA1238 and LGA1239	-
Nectomys squamipes	KY498443	D-loop	LGA2521	-
Nectomys squamipes	KY498444	D-loop	LPC1278	-
Nectomys squamipes	KY498445	D-loop	LPC1283	-
Nectomys squamipes	KY498446	D-loop	LPC1347	-
Nectomys squamipes	KY498447	D-loop	LPC1367 and LPC1375	-
Nectomys squamipes	KY498448	D-loop	MBML2467	-
Nectomys squamipes	KY498449	D-loop	MBML2685	-
Nectomys squamipes	KY498450	D-loop	MCNM303 and MCNM304	-
Nectomys squamipes	KY498451	D-loop	MCNM1362	-
Nectomys squamipes	KY498452	D-loop	MCNM1371	-
Nectomys squamipes	KY498453	D-loop	MCNM1593	-
Nectomys squamipes	KY498454	D-loop	MCNM1920	-
Nectomys squamipes	KY498455	D-loop	MCNM1928	-
Nectomys squamipes	KY498456	D-loop	MCNM1930	-
Nectomys squamipes	KY498457	D-loop	MCNM2005	-
Nectomys squamipes	KY498458	D-loop	MCNM2045	-
Nectomys squamipes	KY498459	D-loop	ML47	-
Nectomys squamipes	KY498460	D-loop	ML61	-
Nectomys squamipes	KY498461	D-loop	ML67 and ML83	-
Nectomys squamipes	KY498462	D-loop	MP318	-
Nectomys squamipes	KY498464	D-loop	RM02	-
Nectomys squamipes	KY498465	D-loop	RM158	-
Nectomys squamipes	KY498466	D-loop	RM167	-
Nectomys squamipes	KY498467	D-loop	RM202	-
Nectomys squamipes	KY498468	D-loop	RM212 and YL793	-
Nectomys squamipes	KY498469	D-loop	ROD135	-
Nectomys squamipes	KY498470	D-loop	ROD131; ROD150 and ROD297	-
Nectomys squamipes	KY498471	D-loop	TAX07; TAX18 and TAX26	-
Nectomys squamipes	KY498472	D-loop	TAX17	-
Nectomys squamipes	KY498473	D-loop	YL774	-
Nectomys squamipes	KY498474	D-loop	YL788	-
Nectomys squamipes	KY498475	D-loop	YL792; YL794 and YL833	-
Nectomys squamipes	KY498476	D-loop	YL806	-
Nectomys squamipes	KY498463	D-loop	MP331	-
Nectomys rattus	KY498404	D-loop	CTA1348	-

Species	Genbank Accession Number	Marker	Sample ID	Haplotypes
Nectomys rattus	**KY498405**	**D-loop**	**CTA1366**	-
Nectomys rattus	**KY498406**	**D-loop**	**UFRO583**	-
Holochilus chacarius	AY863421	D-loop	-	-
Oligoryzomys chacoensis	GU185872	D-loop	Or22498	-
Oligoryzomys flavescens	GU185879	D-loop	UP51	-
Oligoryzomys nigripes	GU185869	D-loop	LIF122	-
Pseudoryzomys simplex	AY863422	D-loop	-	-

Table S2.4: List of size alleles for all six microsatellites loci for 152 samples used in this work organized alphabetically by municipalities. ? = missing data. SeA = Southeast Atlantic; P = Paraná; EA = East Atlantic; SF = São Francisco; SA = South Atlantic; BA = Bahia; ES = Espírito Santo; MG = Minas Gerais; RJ = Rio de Janeiro, and SP = São Paulo.

Sample ID	Municipality (UF)	Basin	Nec 14		Nec 12		Nec 15		Nec 18		Nec 19		Nec 23	
CTA128	Águia Branca (ES)	SeA	232	232	?	?	194	206	?	?	?	?	351	351
CTA135	Águia Branca (ES)	SeA	222	230	?	?	200	206	?	?	229	235	363	365
CTA238	Águia Branca (ES)	SeA	210	228	226	228	198	202	131	127	231	237	381	383
CTA239	Águia Branca (ES)	SeA	224	226	216	218	200	202	157	169	233	233	351	355
CTA240	Águia Branca (ES)	SeA	208	224	216	226	200	202	139	141	233	245	355	357
MP318	Aiuruoca (MG)	P	212	220	210	216	198	200	143	151	239	245	353	357
MBML2306	Anchieta (ES)	SeA	198	212	230	232	194	194	137	141	237	239	453	453
LPC1347	Aracruz (ES)	SeA	216	224	222	228	200	202	133	143	227	239	359	359
MCNM1425	Barão de Cocais (MG)	SeA	204	226	212	218	168	168	137	139	231	237	359	365
MCNM2458	Barbacena (MG)	P	226	226	232	232	198	198	137	157	?	?	353	355
RM167	Cairu (BA)	EA	190	190	?	?	190	210	163	163	?	?	381	383
ROD131	Cananeia (SP)	SA	210	210	212	218	186	190	131	131	233	245	361	361
ROD135	Cananeia (SP)	SA	220	220	212	214	186	194	125	131	239	245	361	375
ROD150	Cananeia (SP)	SA	220	220	212	212	192	196	131	131	241	277	349	355
ROD152	Cananeia (SP)	SA	210	220	214	224	186	196	125	131	241	241	341	359
ROD154	Cananeia (SP)	SA	212	220	214	214	194	196	131	153	235	235	361	381
ROD297	Cananeia (SP)	SA	216	222	214	216	192	194	125	127	235	235	355	361
CTA388	Cariacica (ES)	SeA	222	230	222	234	176	194	151	159	213	233	359	377
CTA508	Cariacica (ES)	SeA	208	216	218	220	198	202	127	135	227	243	379	447
CTA509	Cariacica (ES)	SeA	208	222	218	232	198	204	145	155	225	229	351	373
CTA510	Cariacica (ES)	SeA	208	220	218	226	202	204	135	145	225	243	373	447
CTA511	Cariacica (ES)	SeA	224	226	212	230	198	202	157	157	225	229	357	379
CTA512	Cariacica (ES)	SeA	198	200	224	224	176	208	137	141	225	283	357	379
CTA513	Cariacica (ES)	SeA	198	200	224	224	176	198	145	167	225	225	379	381
CTA514	Cariacica (ES)	SeA	208	208	218	232	198	202	?	?	229	243	373	447
CTA515	Cariacica (ES)	SeA	208	222	?	?	176	198	?	?	217	217	353	377
CTA516	Cariacica (ES)	SeA	222	226	216	230	170	170	127	141	225	225	359	381
CTA517	Cariacica (ES)	SeA	208	228	226	230	168	204	145	155	217	229	359	371
CTA518	Cariacica (ES)	SeA	220	222	220	224	192	198	155	155	233	283	359	379
MBML2685	Castelo (ES)	SeA	200	220	224	224	166	194	?	?	237	243	369	443
LPC1278	Conceição da Barra (ES)	EA	222	222	222	222	202	204	153	153	235	243	367	369
LPC1289	Conceição da Barra (ES)	EA	220	230	222	226	200	202	127	135	229	229	375	391
LPC1291	Conceição da Barra (ES)	EA	204	224	214	222	194	206	133	135	229	231	363	369
LPC1295	Conceição da Barra (ES)	EA	206	208	220	224	200	202	141	141	223	235	363	365
LPC1297	Conceição da Barra (ES)	EA	208	224	216	220	200	206	147	147	217	223	365	387
LPC1300	Conceição da Barra (ES)	EA	206	224	216	234	188	188	141	153	217	235	347	387

Sample ID	Municipality (UF)	Basin	Nec 14		Nec 12		Nec 15		Nec 18		Nec 19		Nec 23	
MCNM2005	Felixlândia (MG)	SF	216	220	208	232	202	204	127	141	?	?	375	457
LGA1215	Ibitirama (ES)	SeA	218	218	?	?	168	168	?	?	231	241	351	381
LGA1238	Ibitirama (ES)	SeA	214	216	224	224	168	168	129	145	239	241	335	381
LGA1239	Ibitirama (ES)	SeA	212	216	224	232	192	206	129	145	233	233	351	367
ML47	Ilhabela (SP)	SeA	200	214	220	222	194	194	129	149	215	239	361	361
RM212	Itamaraju (BA)	EA	228	230	?	?	188	202	145	157	229	231	371	383
RM202	Itamari (BA)	EA	222	228	222	224	186	186	129	147	223	231	385	441
CIT1574	Jussari (BA)	EA	222	226	224	224	194	196	155	155	221	235	351	375
CIT1586	Jussari (BA)	EA	200	200	216	218	196	200	161	167	243	243	353	373
CIT1596	Jussari (BA)	EA	204	230	?	?	202	204	?	?	219	219	365	377
CIT1604	Jussari (BA)	EA	204	204	?	?	164	206	?	?	219	219	365	365
ML67	Lagoa Santa (MG)	SF	200	222	226	236	184	194	141	157	213	229	321	385
ML83	Lagoa Santa (MG)	SF	200	214	214	220	196	202	127	155	225	245	357	367
TAX17	Linhares (ES)	SeA	206	208	226	230	198	200	151	153	231	235	377	379
TAX18	Linhares (ES)	SeA	216	218	224	224	194	202	141	147	239	247	349	357
TAX26	Linhares (ES)	SeA	216	218	226	228	196	206	133	133	229	241	373	451
TAX7	Linhares (ES)	SeA	206	208	206	220	194	198	159	159	229	231	343	375
CTA1119	Marliéria (MG)	SeA	212	224	212	224	194	206	127	127	231	231	349	377
MCNM1920	Nova Lima (MG)	SF	212	226	210	232	200	202	127	163	223	243	333	333
MCNM1928	Nova Lima (MG)	SF	218	226	210	232	198	200	127	159	223	235	335	335
MCNM1930	Nova Lima (MG)	SF	218	226	224	232	200	202	127	127	223	237	333	355
CTA74	Nova Viçosa (BA)	EA	224	232	220	226	204	206	143	157	?	?	363	373
CTA75	Nova Viçosa (BA)	EA	200	204	212	224	204	206	155	157	233	235	355	357
CTA756	Nova Viçosa (BA)	EA	222	222	?	?	196	206	135	143	?	?	355	373
CTA757	Nova Viçosa (BA)	EA	226	232	224	226	206	208	135	155	235	235	373	381
CTA76	Nova Viçosa (BA)	EA	220	222	218	226	196	206	135	165	235	241	355	363
CTA77	Nova Viçosa (BA)	EA	198	222	220	222	196	204	149	157	235	235	343	379
CTA78	Nova Viçosa (BA)	EA	222	224	220	224	202	210	151	151	239	243	371	459
CTA79	Nova Viçosa (BA)	EA	212	222	218	218	194	196	143	147	?	?	371	373
CTA80	Nova Viçosa (BA)	EA	224	224	224	226	168	168	135	135	219	235	357	363
CTA81	Nova Viçosa (BA)	EA	232	232	222	226	180	180	135	157	235	289	365	375
CTA82	Nova Viçosa (BA)	EA	226	232	222	222	202	204	137	157	219	235	347	377
CTA83	Nova Viçosa (BA)	EA	220	226	222	232	202	206	147	169	229	241	351	373
CTA84	Nova Viçosa (BA)	EA	220	230	224	232	202	206	103	147	233	239	369	371
CTA89	Nova Viçosa (BA)	EA	220	224	208	226	180	194	145	147	219	227	345	353
CTA90	Nova Viçosa (BA)	EA	222	224	224	226	168	180	135	145	219	235	351	357
CTA91	Nova Viçosa (BA)	EA	218	222	208	228	176	176	135	155	219	233	347	375
CTA93	Nova Viçosa (BA)	EA	222	222	218	226	196	206	147	157	235	243	363	373
CTA94	Nova Viçosa (BA)	EA	204	232	224	226	204	206	135	155	235	235	355	373
MCNM1371	Ouro Preto (MG)	SeA	218	218	?	?	206	208	127	149	239	255	381	383
ISSP1	Pancas (ES)	SeA	224	224	210	212	168	206	163	165	229	255	389	391
ISSP2	Pancas (ES)	SeA	210	226	210	212	206	208	129	141	245	255	369	371
ISSP3	Pancas (ES)	SeA	224	226	212	222	196	200	141	165	233	245	361	363

Sample ID	Municipality (UF)	Basin	Nec 14		Nec 12		Nec 15		Nec 18		Nec 19		Nec 23	
ISSP4	Pancas (ES)	SeA	206	224	212	228	202	204	143	149	231	233	369	371
ISSP5	Pancas (ES)	SeA	210	210	?	?	200	200	?	?	229	245	371	393
ISSP8	Pancas (ES)	SeA	218	226	212	224	200	202	163	165	229	245	363	365
ISSP9	Pancas (ES)	SeA	218	224	210	212	168	168	149	165	229	233	359	391
FER01	Presidente Kennedy (ES)	SeA	202	214	210	224	198	200	143	163	229	233	367	389
CTA925	Santa Bárbara (MG)	SeA	222	228	224	224	198	202	139	147	233	237	345	369
MCNM1362	Santa Cruz do Escalvado (MG)	SeA	210	212	222	232	204	208	137	159	233	233	367	377
CTA803	Santa Teresa (ES)	SeA	208	222	220	220	188	196	?	?	?	?	365	383
LGA140	Santa Teresa (ES)	SeA	208	216	?	?	204	206	131	131	231	235	379	451
LGA151	Santa Teresa (ES)	SeA	208	224	224	224	192	202	151	157	233	233	337	337
LGA85	Santa Teresa (ES)	SeA	208	220	?	?	206	208	141	157	229	239	349	351
LGA95	Santa Teresa (ES)	SeA	204	216	226	226	198	200	?	?	233	235	371	379
LGA96	Santa Teresa (ES)	SeA	208	226	212	212	200	202	135	135	223	233	355	377
MBML2467	Santa Teresa (ES)	SeA	216	216	?	?	204	208	135	157	?	?	375	375
MP331	Santo Antônio do Amparo (MG)	P	222	228	210	216	188	200	153	159	217	217	357	375
CTA1108	São Gonçalo do Rio Preto (MG)	EA	216	222	210	222	200	206	141	151	237	241	359	359
CTA928	São Gonçalo do Rio Preto (MG)	EA	216	228	226	230	184	208	141	141	229	231	367	371
CTA930	São Gonçalo do Rio Preto (MG)	EA	204	204	212	220	204	212	137	141	223	223	367	431
LPC1367	São José do Calçado (ES)	SeA	210	218	208	208	182	190	145	147	217	231	351	361
LPC1375	São José do Calçado (ES)	SeA	188	200	208	208	208	210	157	159	231	233	353	361
LPC1391	São José do Calçado (ES)	SeA	208	232	208	224	196	196	145	163	239	241	361	377
LPC1392	São José do Calçado (ES)	SeA	188	198	222	230	206	208	145	163	235	239	363	365
ROD41	São Luís do Paraitinga (SP)	SeA	220	224	214	220	182	192	129	143	237	237	347	347
LGA2521	São Mateus (ES)	EA	226	228	214	232	192	192	143	153	231	233	333	369
YL774	Serra (ES)	SeA	214	236	212	226	202	204	141	141	219	225	337	353
CTA1737	Sorocaba (SP)	P	222	222	214	216	186	204	?	?	225	225	373	373
CTA1738	Sorocaba (SP)	P	216	222	214	214	186	202	151	159	241	293	363	365
CTA1739	Sorocaba (SP)	P	212	222	214	214	202	204	125	157	225	235	371	373
CTA1745	Sorocaba (SP)	P	220	222	210	214	192	202	?	?	?	?	?	?
CTA1746	Sorocaba (SP)	P	222	222	214	218	200	202	?	?	?	?	363	367
CTA1747	Sorocaba (SP)	P	216	222	?	?	198	202	?	?	?	?	357	381
CTA1751	Sorocaba (SP)	P	212	212	214	214	202	204	125	157	225	235	369	371
CTA1757	Sorocaba (SP)	P	212	222	214	214	194	202	125	127	?	?	369	387
LPC857	Sorocaba (SP)	P	204	208	210	214	190	202	157	157	227	239	369	369
LBCE7781	Sumidouro (RJ)	SeA	220	222	234	234	202	204	163	165	231	231	351	351
LBCE7805	Sumidouro (RJ)	SeA	206	222	216	224	186	200	143	143	231	239	353	369
AB598	Tapiraí (SP)	P	220	232	214	220	196	202	129	135	233	239	343	357
MCNM303	Teixeiras (MG)	SeA	202	224	214	214	194	208	127	145	237	241	377	379
MCNM304	Teixeiras (MG)	SeA	204	220	212	214	194	196	127	163	239	241	347	347
ML61	Ubatuba (SP)	SeA	216	222	212	224	198	220	127	143	229	231	365	373
CTA968	Una (BA)	EA	212	228	?	?	194	198	?	?	231	239	361	379
YL784	Una (BA)	EA	210	216	220	222	194	210	137	139	231	231	359	445

Sample ID	Municipality (UF)	Basin	Nec 14		Nec 12		Nec 15		Nec 18		Nec 19		Nec 23	
YL788	Una (BA)	EA	210	228	214	224	188	200	145	161	217	229	359	379
YL792	Una (BA)	EA	202	228	212	222	200	208	147	153	219	227	371	379
YL793	Una (BA)	EA	204	228	230	230	198	200	127	127	229	229	353	357
YL794	Una (BA)	EA	208	210	220	222	196	198	143	155	229	237	351	353
YL806	Una (BA)	EA	216	224	224	224	206	208	147	147	231	231	357	359
YL807	Una (BA)	EA	226	226	212	230	196	202	153	155	231	231	355	359
YL817	Una (BA)	EA	226	228	220	224	194	200	149	149	221	231	369	379
YL833	Una (BA)	EA	226	228	230	230	198	210	147	155	217	229	351	369
CTA616	Viana (ES)	SeA	212	228	?	?	198	200	?	?	?	?	355	387
CTA620	Viana (ES)	SeA	204	220	?	?	176	208	131	145	225	229	365	447
CTA627	Viana (ES)	SeA	206	222	210	216	198	200	?	?	?	?	379	379
CTA719	Viana (ES)	SeA	210	212	214	220	196	198	129	145	229	231	387	447
CTA720	Viana (ES)	SeA	212	222	?	?	198	206	?	?	225	229	375	389
CTA721	Viana (ES)	SeA	222	222	210	216	198	208	147	147	225	235	387	389
CTA722	Viana (ES)	SeA	210	224	218	224	198	200	145	145	225	227	357	365
CTA723	Viana (ES)	SeA	210	210	218	224	198	200	?	?	225	229	345	365
CTA724	Viana (ES)	SeA	210	222	210	216	176	208	127	147	237	243	387	447
CTA725	Viana (ES)	SeA	208	224	224	228	198	200	145	145	227	235	355	377
CTA726	Viana (ES)	SeA	208	210	226	228	200	206	147	147	229	229	341	365
CTA727	Viana (ES)	SeA	222	230	218	230	196	198	127	157	225	243	361	373
CTA728	Viana (ES)	SeA	212	212	?	?	176	188	?	?	?	?	361	377
CTA729	Viana (ES)	SeA	208	228	232	232	188	192	?	?	237	283	349	357
CTA730	Viana (ES)	SeA	198	224	228	232	188	198	133	135	215	227	377	383
CTA731	Viana (ES)	SeA	208	212	?	?	168	202	127	127	213	233	351	387
CTA733	Viana (ES)	SeA	206	210	210	238	200	202	145	145	235	243	379	381
CTA734	Viana (ES)	SeA	206	226	210	210	198	206	127	127	215	243	361	381
CTA735	Viana (ES)	SeA	206	228	210	210	200	206	141	141	243	243	359	361
CTA736	Viana (ES)	SeA	212	224	210	220	176	202	?	?	225	225	341	373
CTA737	Viana (ES)	SeA	208	224	226	230	196	198	127	143	229	243	353	387
CTA858	Viana (ES)	SeA	210	224	210	218	198	208	145	147	?	?	345	449
RM158	Wenceslau Guimarães (BA)	EA	206	224	230	236	202	206	137	139	237	237	371	373

Table S2.5: Deviations from Hardy-Weinberg equilibrium (HWE) in global tests per locus and for all loci and all populations, and the average frequencies of null alleles per locus. Mean frequency of null alleles for each locus was always below 0.14.

	**HWE**	**Null Alleles**
Nec12	0.08	0.08
Nec14	0.98	0.06
Nec15	0.94	0.03
Nec18	0.19	0.10
Nec19	0.46	0.08
Nec23	0.87	0.04
All loci	0.79	-

Table S2.6: Basic descriptive statistics for the six microsatellite loci separately and the average of them for the species *Nectomys squamipes*. N=number of alleles; Ma= median alleles; Ho= Observed heterozigosity; He= Expected heterozigosity; Hs= Gene diversity; Rs= Allelic richness; Ap= Frequency of private alleles.

Microsatellite marker	Genetic index	Paraná	South Atlantic	Southeast Atlantic	East Atlantic	São Francisco	Total
**Nec 12** *Range: 206-244*	*N*	6	5	16	15	8	17
	Ho	0.58	0.67	0.74	0.8	1	0.82
	He	0.68	0.76	0.92	0.89	0.89	0.92
	Hs	0.69	0.77	0.92	0.9	0.88	0.92
	Rs	4.23	4.5	7.11	6.55	6.99	7.1
**Nec 14** *Range: 188-236*	*N*	9	5	20	18	8	21
	Ho	0.62	0.5	0.87	0.78	1	0.77
	He	0.83	0.73	0.93	0.92	0.92	0.93
	Hs	0.82	0.75	0.93	0.92	0.92	0.93
	Rs	5.52	4.5	7.49	7.24	7.14	7.47
**Nec 15** *Range: 264-220*	*N*	10	5	19	19	7	23
	Ho	0.92	1	0.89	0.87	1	0.89
	He	0.85	0.85	0.91	0.92	0.86	0.92
	Hs	0.85	0.83	0.91	0.93	0.85	0.92
	Rs	6	4.82	6.94	7.32	6.17	7.15
**Nec 18** *Range: 103-187*	*N*	10	4	21	21	6	24
	Ho	0.89	0.67	0.74	0.74	0.83	0.76
	He	0.9	0.64	0.93	0.93	0.76	0.94
	Hs	0.9	0.63	0.94	0.93	0.75	0.94
	Rs	6.89	3.67	7.6	7.53	5.32	7.83
**Nec 19** *Range: 213-277*	*N*	9	6	19	14	8	23
	Ho	0.75	0.5	0.82	0.69	1	0.78
	He	0.91	0.85	0.92	0.9	0.93	0.93
	Hs	0.92	0.88	0.92	0.9	0.93	0.93
	Rs	6.83	5.49	7.13	6.63	8	7.35
**Nec 23** *Range: 321-459*	*N*	13	7	33	28	9	41
	Ho	0.83	0.83	0.88	0.96	0.67	0.89
	He	0.93	0.83	0.96	0.96	0.94	0.96
	Hs	0.94	0.83	0.96	0.96	0.97	0.96
	Rs	7.56	6.15	8.5	8.3	7.82	8.5
**Mean**	*n*	13	6	82	46	6	153
	Ma	9.5	5.33	21.33	19.17	7.67	24.83
	Ho	0.77	0.69	0.82	0.81	0.92	0.81
	He	0.85	0.78	0.93	0.92	0.89	0.93
	Hs	0.85	0.78	0.93	0.92	0.88	0.93
	Rs	6.17	4.85	7.46	7.26	6.9	7.57
	Ap	-	-	-	-	-	0.02

Table S2.7: Genetic distances (p-distance) for Cyt b (italics values), and D-loop (bold values) between and within (diagonal) the river basins, mtDNA clades, and BAPS clusters. Some BAPS clusters were not recovered for both markers, see text to details. NA: not applicable due to lack of samples.

		São Francisco basin	Paraná basin	South Atlantic basin	Southeast Atlantic basin	East Atlantic basin	Paraguay basin
River Basins	**São Francisco basin**	*0.016* – **0.039**	**0.032**	**0.03**	**0.04**	**0.039**	**NA**
	**Paraná basin**	*0.017*	*0.012* – **0.019**	**0.019**	**0.04**	**0.039**	**NA**
	**South Atlantic basin**	*0.016*	*0.013*	*0* – **0.005**	**0.043**	**0.041**	**NA**
	**Southeast Atlantic basin**	*0.019*	*0.02*	*0.021*	*0.01* – **0.018**	**0.024**	**NA**
	**East Atlantic basin**	*0.017*	*0.02*	*0.021*	*0.011*	*0.009* – **0.022**	**NA**
	**Paraguay basin**	*0.012*	*0.013*	*0.009*	*0.018*	*0.018*	**NA**

		North clade	Central clade	South clade
Clades	**North clade**	*0.005* – **0.016**	**0.040**	**0.043**
	**Central clade**	*0.018*	*0.006* – **0.017**	**0.049**
	**South clade**	*0.019*	*0.018*	*0.008* – **0.015**

		North cluster	Central cluster	South cluster	South Atlantic cluster	Caparaó cluster	North Doce River cluster	South Doce River cluster
BAPS Clusters	**North cluster**	*0.005* – **0.016**	-	**0.043**	**0.0381**	**0.0386**	**0.043**	**0.0414**
	**Central cluster**	0.018	*0.006* –	**0.049**	-	-	-	-
	**South cluster**	*0.019*	*0.018*	*0.008* – **0.015**	**0.0431**	**0.0383**	**0.0436**	**0.0514**
	**South Atlantic cluster**	-	-	-	– **0.0107**	**0.0236**	**0.0182**	**0.0196**
	**Caparaó cluster**	-	-	-	-	– **0.0111**	**0.0277**	**0.0332**
	**North Doce River cluster**	-	-	-	-	-	– **0.0021**	**0.0276**
	**South Doce River cluster**	-	-	-	-	-	-	– **0.0045**

Table S2.8: Dates (millions of years ago) of major cladogenesis for Cyt b related to *Nectomys,* and corresponding confidence intervals.

Cladogenesis	Median	Confidence Interval (95 %)	
		**Upper**	**Lower**
Origin of *Nectomys*	1.99	1.30	2.70
Nectomys rattus + *Nectomys squamipes*	1.35	0.72	2.05
Origin of *Nectomys rattus*	0.82	0.35	1.48
Origin of *Nectomys squamipes*	1.01	0.51	1.64
South clade	0.53	0.19	1.03
North clade + Central clade	0.88	0.43	1.44
North clade	0.48	0.15	0.95
Central clade	0.65	0.29	1.15

Table S2.9: Molecular diversity indices and neutrality tests for *Nectomys squamipes*. Populations organized by basins, clades and BAPS clusters for each genetic marker. Significant values are in bold. *n* = sample size; H = number of haplotypes; S = number of segregating sites; Hd = haplotype diversity; Pi = nucleotide diversity; Fs = Fu's Fs; R_2 = R_2 test; D = Tajima's D; p = p-values.

	Basins / Clades / Clusters	*n*	H	S	Hd	Pi	Fs	p	R_2	p	D	p
Cyt b	São Francisco basin	4	3	18	0.833	0.014	2.81	0.85	0.26	0.58	1.4	0.89
	Paraná basin	8	4	17	0.643	0.007	1.67	0.8	0.2	0.8	-0.87	0.20
	South Atlantic basin	3	1	0	0	0	–	–	–	–	–	–
	Paraguay basin	1	1	–	–	–	–	–	–	–	–	–
	Southeast Atlantic basin	38	18	36	0.841	0.006	**-6.18**	**0.01**	**0.11**	**0.03**	**-1.76**	**0.01**
	East Atlantic basin	23	11	30	0.806	0.009	0.31	0.58	0.11	0.35	0.05	0.36
	South clade / cluster	14	7	19	0.846	0.008	1.08	0.7	0.14	0.52	0.09	0.57
	Central clade / cluster	55	22	31	0.885	0.005	**-9.45**	**<0.01**	**0.05**	**0.02**	**-1.48**	**0.04**
	North clade / cluster	8	7	13	0.964	0.005	-2.37	0.05	0.13	0.14	-0.88	0.22
	Nectomys squamipes	77	36	63	0.936	0.011	**-10.88**	**0.01**	0.06	0.11	-1.07	0.13
D-loop	São Francisco basin	7	6	38	0.952	0.039	1.05	0.6	0.18	0.5	0.34	0.64
	Paraná basin	12	5	26	0.667	0.017	3.49	0.93	0.12	0.23	-0.66	0.28
	South Atlantic basin	4	2	4	0.500	0.005	2.2	0.82	0.24	1	-0.78	0.22
	Southeast Atlantic basin	81	34	51	0.941	0.018	**-10.53**	**<0.01**	0.09	0.23	-0.8	0.25
	East Atlantic basin	44	24	46	0.942	0.022	-3.88	0.1	0.09	0.35	-0.33	0.39
	South clade / cluster	20	10	28	0.858	0.015	-0.12	0.51	0.12	0.42	-0.81	0.24
	Central clade	118	51	52	0.965	0.017	**-24.49**	**<0.01**	0.07	0.25	0.51	0.27
	North clade / cluster	10	8	21	0.956	0.016	-1.2	0.23	0.13	0.22	-0.4	0.35
	Southeast Atlantic cluster	54	31	39	0.969	0.011	**-20.89**	**<0.01**	**0.05**	**0.01**	**-1.57**	**0.03**
	Caparaó cluster	9	5	12	0.861	0.011	1.07	0.7	0.18	0.62	0.33	0.66
	North Doce River cluster	16	4	3	0.642	0.002	-0.51	0.39	0.15	0.43	-0.04	0.55
	South Doce River cluster	39	11	12	0.795	0.004	-3.62	0.08	0.07	0.14	-0.99	0.16
	Nectomys squamipes	148	69	80	0.975	0.026	**-24.2**	**<0.01**	0.07	0.28	-0.7	0.27

Table S2.10: Heterozygote rates found for each basin and for *Nectomys squamipes*. Deficiency and excess of heterozogote (H) is shown for models of evolution of microsatellites Two-Phased Model (TPM) and Stepwise Mutation Model (SSM). Significant deviations to heterozygote excess indicate a bottleneck event and to heterozygote deficit indicate a population expansion. Non-significant values indicate population stability. n = number of samples; TPM = Two-Phased Model; SSM = Stepwise Mutation Model; H = heterozogote; * = p-value <0.05 (Wilcocox Test).

		**TPM**		**SMM**		
**Basin/species**	**n**	**H deficiency**	**H excess**	**H deficiency**	**H excess**	
Paraná	13	0.156*	0.992	0.156*	0.992	**expansion**
Southeast Atlantic	81	0.781	0.281	0.719	0.344	**stability**
East Atlantic	46	1.000	0.007*	0.500	0.578	**bottleneck**
Nectomys squamipes	152	0.960	0.054	0.500	0.578	**stability**

Table S2.11: F_{ST} and R_{ST} among populations of *Nectomys squamipes* from different basins in the Atlantic Forest. F_{ST} values from Cyt b (below the diagonal) and D-loop (above the diagonal) are in mitochondrial DNA (mtDNA), and R_{ST} values from microsatellite data are showed below the mitochondrial markers. * = p < 0.05; ** = p < 0.01;

	**Basin**	**Paraná**	**East Atlantic**	**South Atlantic**	**Southeast Atlantic**
	Paraná	-	0.17**	0.39**	0.17**
	East Atlantic	0.25**	-	0.20**	0.06**
$\mathbf{F_{ST}}$	South Atlantic	0.55**	0.39**	-	0.19**
	Southeast Atlantic	0.23**	0.17**	0.36**	-
	São Francisco	0.28*	0.18**	0.52*	0.16**
	Paraná	-			
	East Atlantic	0.04**	-		
$\mathbf{R_{ST}}$	South Atlantic	0.10**	0.10**	-	
	Southeast Atlantic	0.04**	0.01**	0.09**	-
	São Francisco	0.05*	0.04**	0.13**	0.03**

Table S2.12: Validation indexes of the species distribution models derived from MaxEnt results and from external validation in bold.

Index	Value
Training samples	49
Test samples	20
Test AUC	0.817
AUC standard deviation	0.04
Minimum training presence logistic threshold	0.14
Minimum training presence test omission	0.02
Minimum training presence binomial probability	<0.01
10 percentile training presence logistic threshold	0.22
10 percentile training presence training omission	0.11
10 percentile training presence binomial probability	<0.01
Maximum test sensitivity plus specificity logistic threshold	0.38
Maximum test sensitivity plus specificity test omission	0.22
Maximum test sensitivity plus specificity binomial probability	<0.01
**Sensitivity**	**0.83**
**Specificity**	**0.58**
**Accuracy**	**0.60**
**True Skill Statistic (TSS)**	**0.40**
**Error of commission**	**0.38**
**Error of omission**	**0.17**
