# Supplementary material for: Historical connections among river basins and climatic changes explain the biogeographic history of a water rat
Source: PeerJ. 2018 Jul 27;6:e5333. doi: 10.7717/peerj.5333 (PMC6065461; doi:10.7717/peerj.5333)
Supplement: Appendix S3 [file peerj-06-5333-s003.pdf]

PeerJ

SUPPORTING INFORMATION
Historical connections among river basins and climatic changes explain the biogeographic history of a water rat

APPENDIX S3: Phylogeny for D-loop and concatenated data (Figure S3.1); phylogeny for Cyt b without *Nectomys apicalis* e *Amphinectomys savamis* (Figure S3.2); haplotype networks for mitochondrial markers (Figure S3.3); probability of occurrence of *Nectomys squamipes* with the increase of the three major variables for the models (Figure S3.4); historical connections among river basins (Fig. S3.5).

Figure S3.1: Phylogeny through Bayesian inference (BI) for D-loop (A) and concatenated data (B). Main clades for *Nectomys squamipes* are represented with white circles, and their bootstrap support for maximum parsimony (MP), maximum likelihood (ML), and the posterior probability for bayesian inference (BI) are also shown in the following order: MP/ML/IB. Clades not recovered in analysis are represented with a dash (-). Central clade was not recovered in D-loop and in IB approach with concatenated data. However, MP and ML with concatenated data recovered Central clade with bootstrap of 93% and 58%, respectively. We chose the BI method because it presented higher values of statistical support. Numbers correspond to localities in Appendix S1 and Fig. 1. The circle colors indicate the river basin where samples occur.

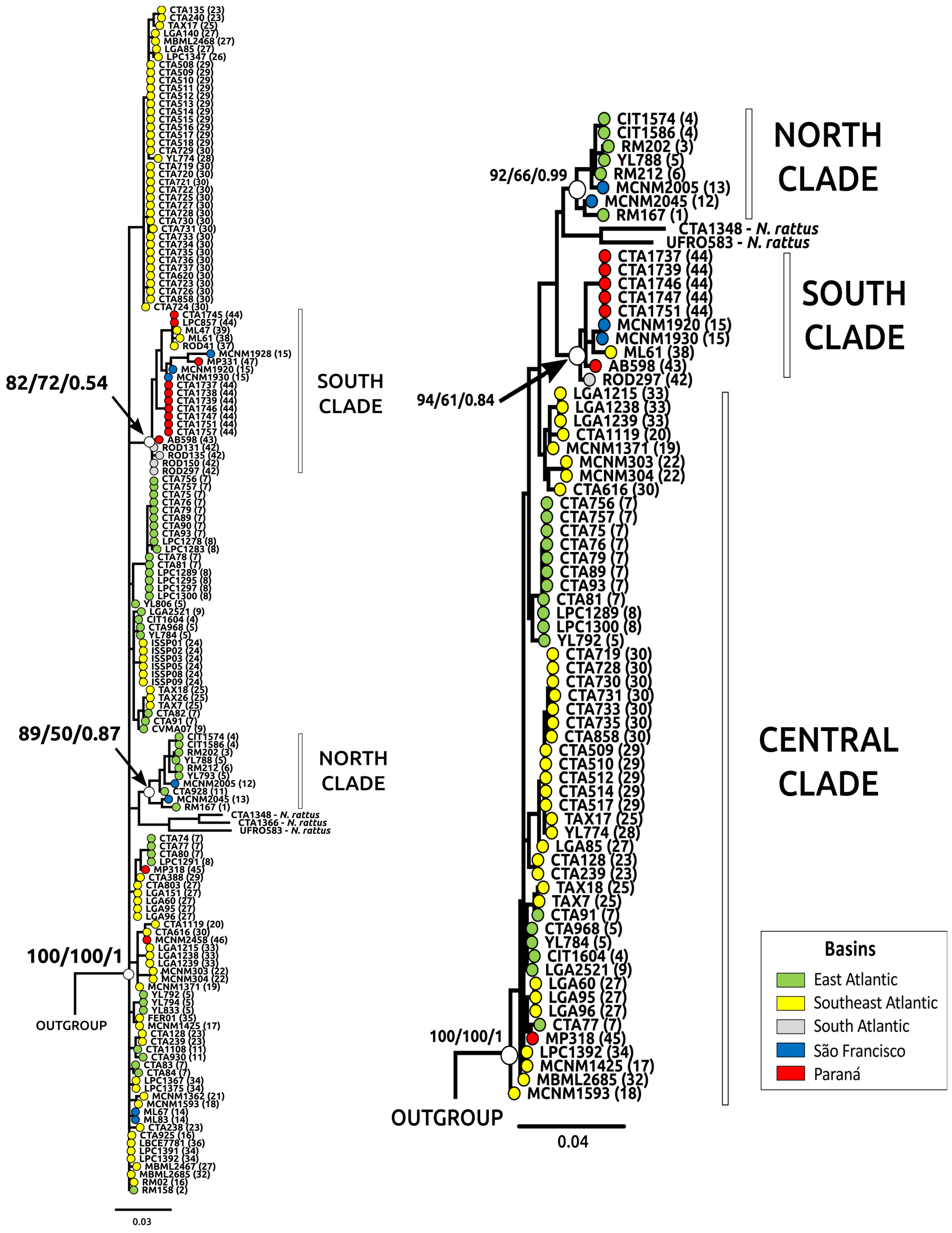
82/72/0.54
SOUTH CLADE
89/50/0.87
NORTH CLADE
100/100/1
OUTGROUP
0.03
CTA1348 - N. rattus
CTA1366 - N. rattus
UFRO583 - N. rattus
NORTH CLADE
92/66/0.99
CIT1574 (4)
CIT1586 (4)
RM202 (3)
YL788 (5)
RM212 (6)
MCNM2005 (13)
MCNM2045 (12)
RM167 (1)
CTA1348 - N. rattus
UFRO583 - N. rattus
SOUTH CLADE
CTA1737 (44)
CTA1739 (44)
CTA1746 (44)
CTA1747 (44)
CTA1751 (44)
MCNM1920 (15)
MCNM1930 (15)
ML61 (38)
AB598 (43)
ROD297 (42)
94/61/0.84
CENTRAL CLADE
LGA1215 (33)
LGA1238 (33)
LGA1239 (33)
CTA1119 (20)
MCNM1371 (19)
MCNM303 (22)
MCNM304 (22)
CTA616 (30)
CTA756 (7)
CTA757 (7)
CTA75 (7)
CTA76 (7)
CTA79 (7)
CTA89 (7)
CTA93 (7)
CTA81 (7)
LPC1289 (8)
LPC1300 (8)
YL792 (5)
CTA719 (30)
CTA728 (30)
CTA730 (30)
CTA731 (30)
CTA733 (30)
CTA735 (30)
CTA858 (30)
CTA509 (29)
CTA510 (29)
CTA512 (29)
CTA514 (29)
CTA517 (29)
TAX17 (25)
YL774 (28)
LGA85 (27)
CTA128 (23)
CTA239 (23)
TAX18 (25)
TAX7 (25)
CTA91 (7)
CTA968 (5)
YL784 (5)
CIT1604 (4)
LGA2521 (9)
LGA60 (27)
LGA95 (27)
LGA96 (27)
CTA77 (7)
MP318 (45)
LPC1392 (34)
MCNM1425 (17)
MBML2685 (32)
MCNM1593 (18)
100/100/1
OUTGROUP
0.04
Basins
East Atlantic
Southeast Atlantic
South Atlantic
São Francisco
Paraná

Figure S3.2: Phylogeny through Bayesian inference (BI) for Cyt *b* without *Nectomys apicalis* e *Amphinectomys savamis* as outgroups recovered a similar topology to D-loop and concatenated data trees. Main clades for *Nectomys squamipes* are represented with white circles, and their bootstrap support for maximum parsimony (MP), maximum likelihood (ML), and the posterior probability for bayesian inference (BI) are also shown in the following order: MP/ML/IB. Clades not recovered in analysis are represented with a dash (-). Central clade was not recovered in IB approach with concatenated data. However, MP and ML recovered Central clade with bootstrap of 85% and 78%, respectively. We chose the BI method because it presented higher values of statistical support. Numbers correspond to localities in Appendix S1 and Fig. 1. The circle colors indicate the river basin where samples occur.

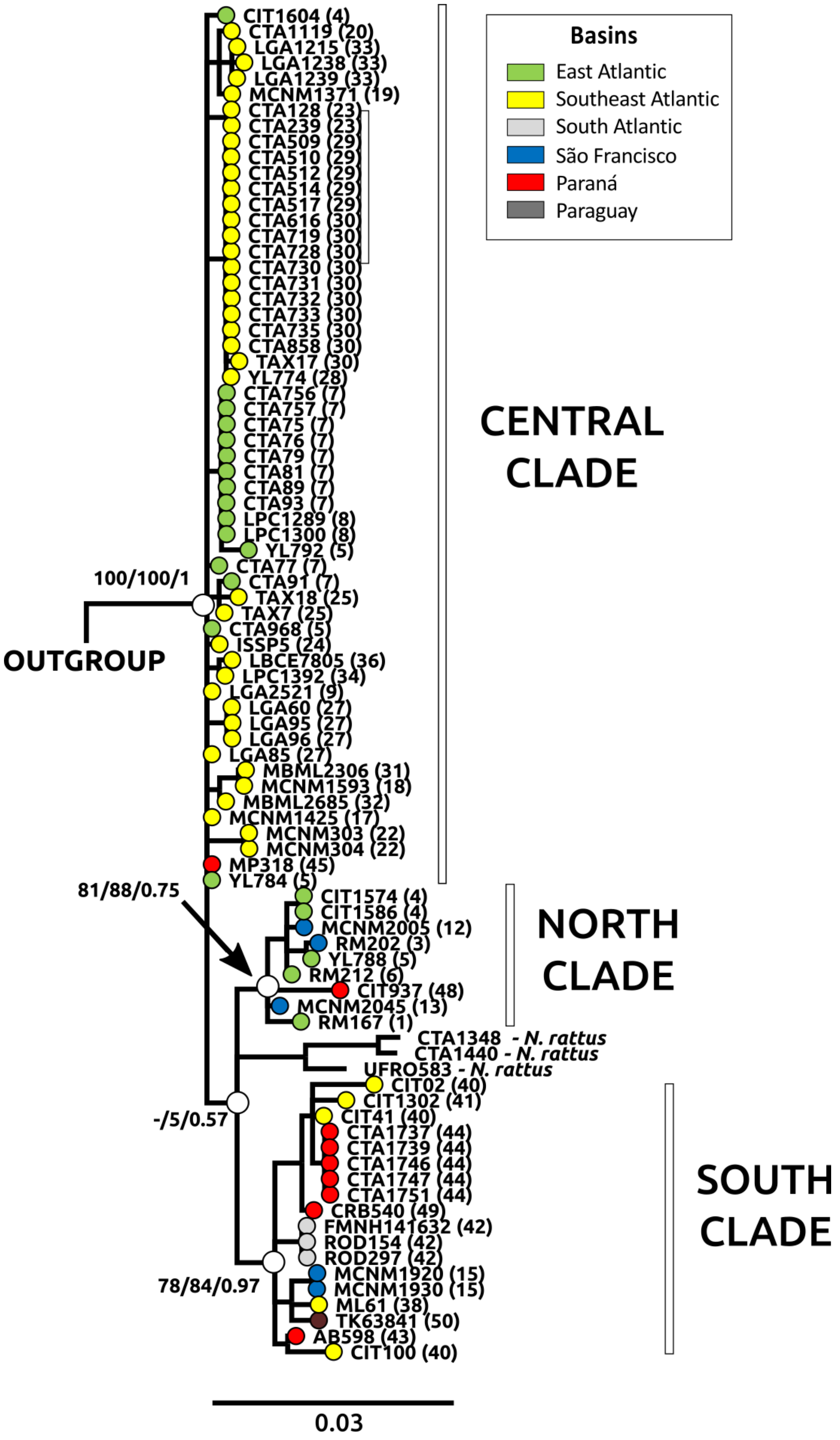
Basins
East Atlantic
Southeast Atlantic
South Atlantic
São Francisco
Paraná
Paraguay
CIT1604 (4)
CTA1119 (20)
LGA1215 (33)
LGA1238 (33)
LGA1239 (33)
MCNM1371 (19)
CTA128 (23)
CTA239 (23)
CTA509 (29)
CTA510 (29)
CTA512 (29)
CTA514 (29)
CTA517 (29)
CTA616 (30)
CTA719 (30)
CTA728 (30)
CTA730 (30)
CTA731 (30)
CTA732 (30)
CTA733 (30)
CTA735 (30)
CTA858 (30)
TAX17 (30)
YL774 (28)
CTA756 (7)
CTA757 (7)
CTA75 (7)
CTA76 (7)
CTA79 (7)
CTA81 (7)
CTA89 (7)
CTA93 (7)
LPC1289 (8)
LPC1300 (8)
YL792 (5)
CTA77 (7)
CTA91 (7)
TAX18 (25)
TAX7 (25)
CTA968 (5)
ISSP5 (24)
LBCE7805 (36)
LPC1392 (34)
LGA2521 (9)
LGA60 (27)
LGA95 (27)
LGA96 (27)
LGA85 (27)
MBML2306 (31)
MCNM1593 (18)
MBML2685 (32)
MCNM1425 (17)
MCNM303 (22)
MCNM304 (22)
MP318 (45)
YL784 (5)
CENTRAL CLADE
100/100/1
OUTGROUP
81/88/0.75
CIT1574 (4)
CIT1586 (4)
MCNM2005 (12)
RM202 (3)
YL788 (5)
RM212 (6)
CIT937 (48)
MCNM2045 (13)
RM167 (1)
NORTH CLADE
CTA1348 - N. rattus
CTA1440 - N. rattus
UFRO583 - N. rattus
-/5/0.57
CIT02 (40)
CIT1302 (41)
CIT41 (40)
CTA1737 (44)
CTA1739 (44)
CTA1746 (44)
CTA1747 (44)
CTA1751 (44)
CRB540 (49)
FMNH141632 (42)
ROD154 (42)
ROD297 (42)
MCNM1920 (15)
MCNM1930 (15)
ML61 (38)
TK63841 (50)
AB598 (43)
CIT100 (40)
78/84/0.97
SOUTH CLADE
0.03

Figure S3.3: Cyt b and D-loop haplotype networks of *Nectomys squamipes*. Colours represent the river basins, the sizes of the circles represent the number of shared haplotypes, the lengths of the lines represent the distance between haplotypes, and the black circles are the mean vectors.

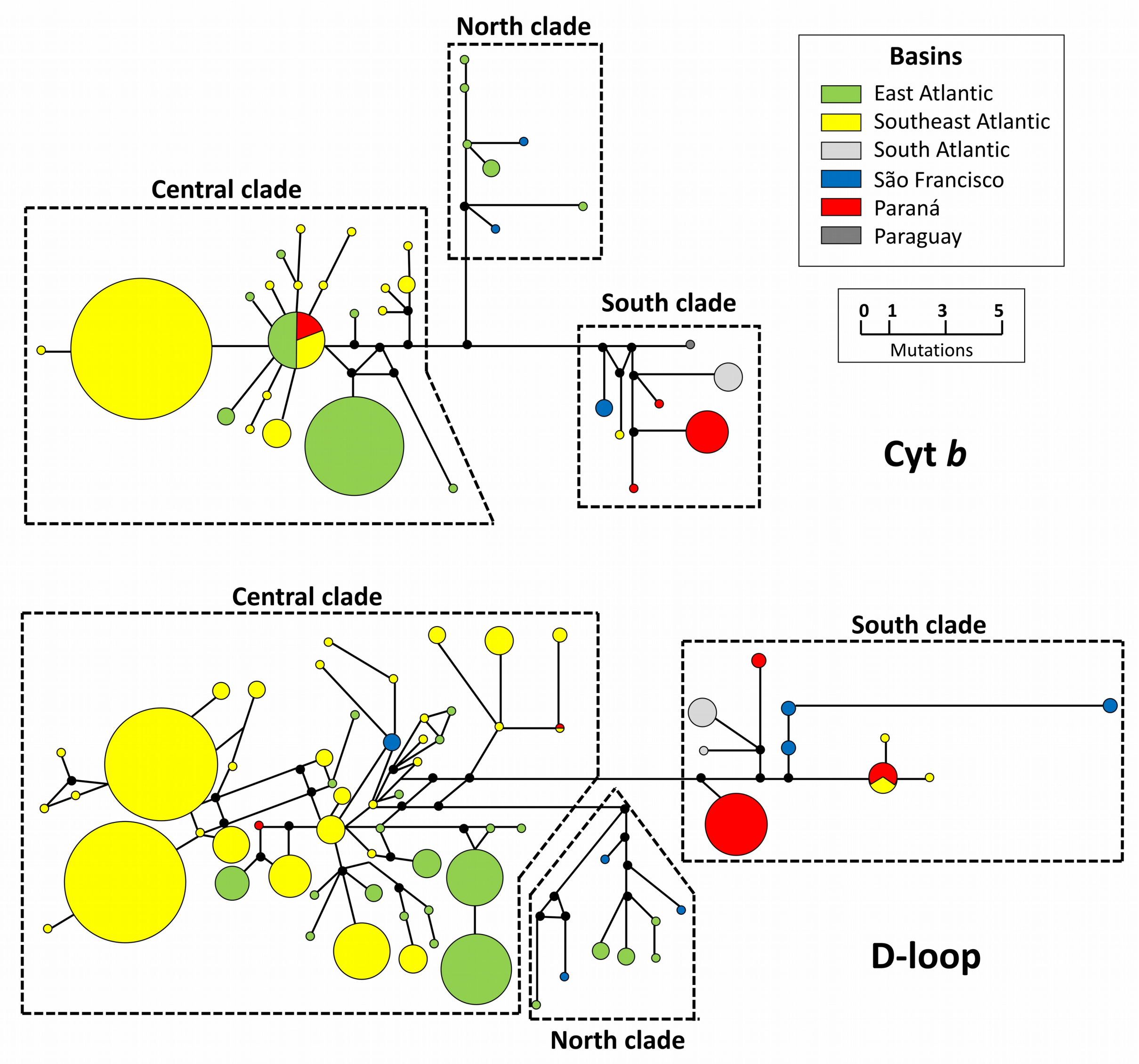

Figure S3.4: Probability of occurrence of *Nectomys squamipes* with the increase of the three major variables for the SDM. Temperature values are multiplied by 10. (a) mean diurnal range - Bio 02; (b) precipitation of warmest quarter - Bio 18; (c) precipitation of wettest month - Bio 13.

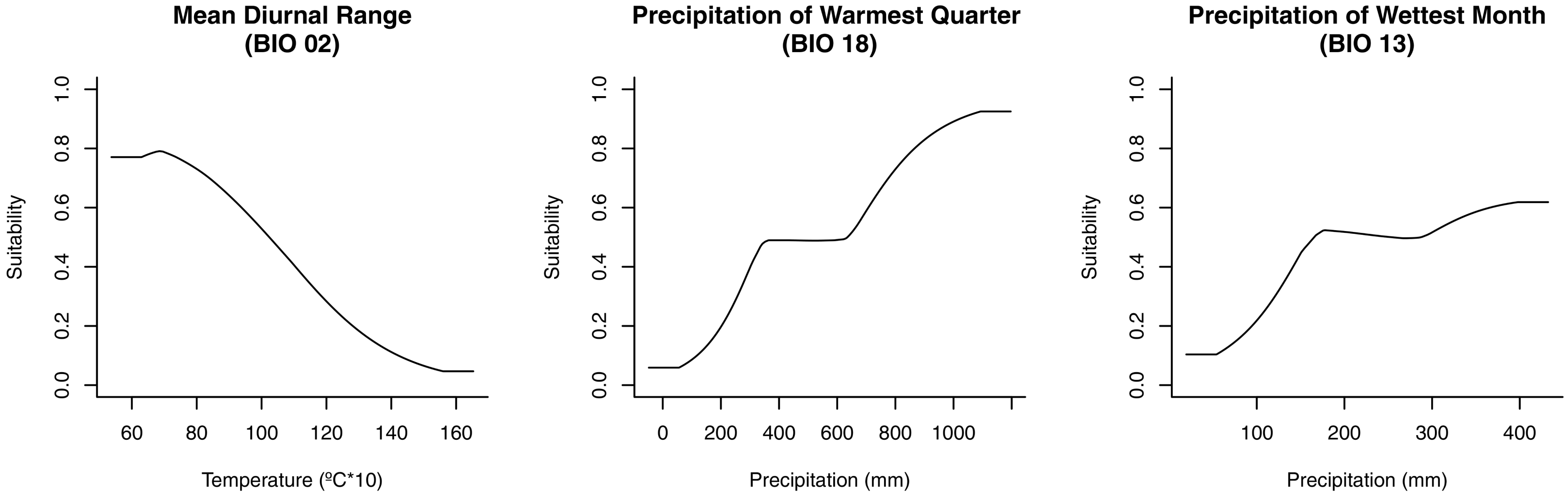

Figure S3.5: Connections among river basins where gene flow was inferred between *Nectomys squamipes* populations. **1**: Southeast Atlantic and East Atlantic basins through the São Francisco River and Doce River tributaries; **2**: Grande River sub-basin (Paraná basin) and the São Francisco and Southeast Atlantic basins; **3**: Grande, Preto, and Paraíba do Sul Rivers; **4**: Tietê and Paraíba do Sul Rivers along the coast of São Paulo and Rio de Janeiro;: Southeast Atlantic and East Atlantic basins through the continental platform exposed during the Last Glacial Maximum (LGM) marine regression.

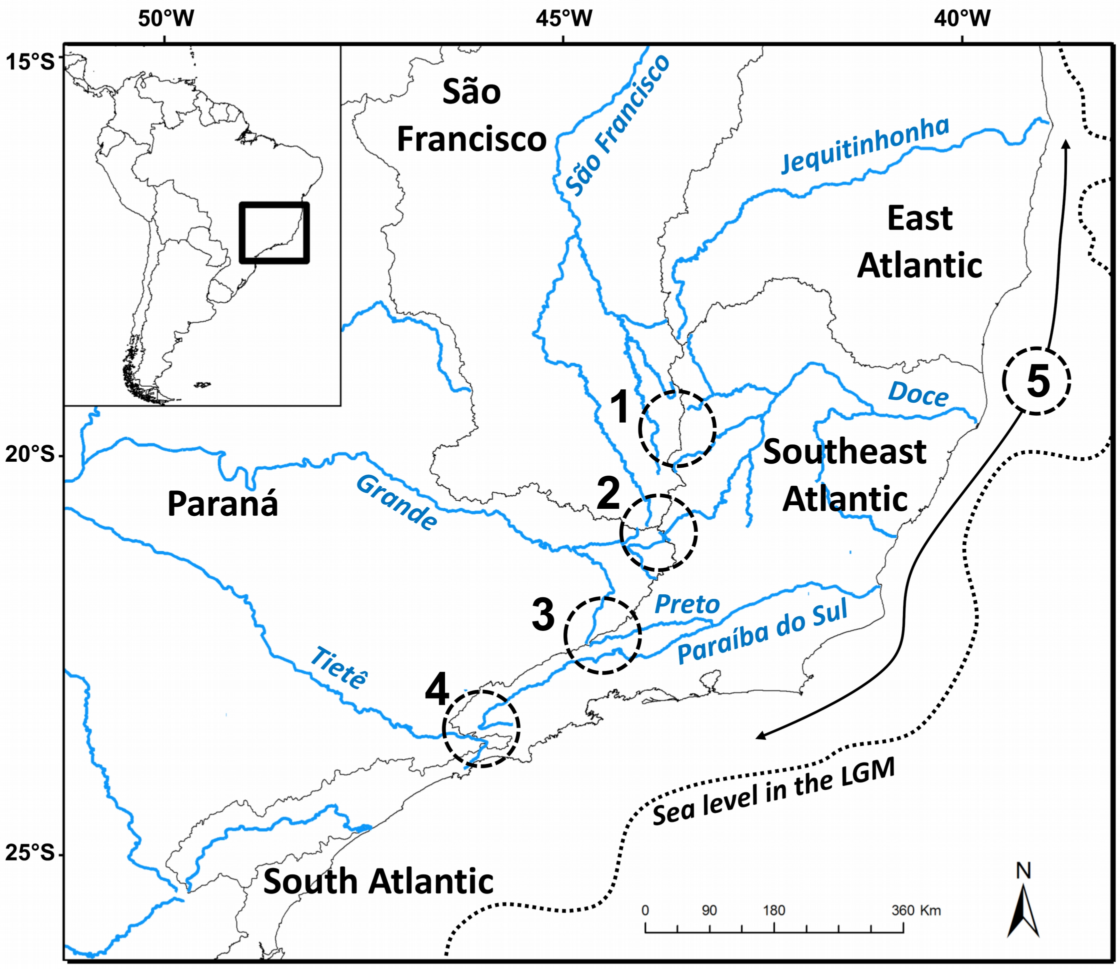

Connection 1 is among the Southeast Atlantic and São Francisco basins between the Doce and Das Velhas rivers, a tributary of the São Francisco river. Vieira *et al.* (2005) counted 12 fish species shared between São Francisco and Southeast Atlantic basins in their study, and the number could be larger if fish fauna of Doce river was better known (Vieira et al., 2005; Vieira, 2010). De Barros *et al.*

(2015) also indicated the connection between Piracicaba river (a tributary of the Doce river) and Das Velhas river as explanation for Cytb haplotypes shared between these different basins in species of the fish genus *Oligosarcus* Günther.

The sub-basin of the Grande River is a key site in the connection among the Paraná, São Francisco and Southeast Atlantic basins (Connection 2). The fish fauna of the São Francisco River is very similar to that of the Paraná River (Menezes, 1970; Buckup, 2011), indicating a historical contact between these two basins. The high number of fish species shared between the two suggests that the link between the Paraná and São Francisco Rivers is geologically recent (Buckup, 2011). These results are in agreement with those found in the present study because the sub-basin of the Grande River has also served as a recent link between the São Francisco and Paraná populations of *N. squamipes*. Moreover, the Grande River sub-basin allowed gene flow between *N. squamipes* populations through the sub-basin of the Doce and/or Preto rivers (Connections 2 and 3). These connections have been previously reported both for fish (Ribeiro, 2006) and rattlesnakes (Bastos *et al.*, 2005).

The connections among the Paraná, South Atlantic, and Southeast Atlantic basins occurred through the upper Tietê and Paraíba do Sul rivers (Connection 4). The Paraíba do Sul and the upper Paraná river basins were connected in the past though the upper Tietê (Ab'Saber, 1957; Ribeiro, 2006; Ingenito & Buckup, 2007), and fossil data indicate that this connection is contemporary with the Tremembé Formation in the Late Oligocene or Early Miocene (Gallego & Mesquita, 2000; Vucetich & Ribeiro, 2003; Ribeiro, 2006), long before the origin of *N. squamipes*. However other connections have been proposed for fishes during the Quaternary (Ribeiro *et al.*, 2006; Serra *et al.*, 2007; Buckup, 2011), and *N. squamipes* could have used these recent connections for dispersal across basins.

The connection between the Southeast Atlantic and the East Atlantic (Connection 5) basins may have been mostly influenced by the fluctuations in sea level during the Quaternary. Ocean regressions are frequently called upon to explain the widespread distribution of some fish species throughout the rivers in the coastal region of the Atlantic Forest (Weitzman *et al.*, 1988; Ribeiro, 2006; Menezes *et al.*, 2008; Buckup, 2011) and some phylogeographic patterns of vertebrates (Grazziotin *et al.*, 2006;

Fitzpatrick *et al.*, 2009). The fish fauna shared among the rivers in these basins are significant, confirming a recent connection (Ribeiro, 2006).

REFERENCES:

Ab'Saber N.A. (1957) O problema das conexões antigas e da separação da drenagem do Paraíba e Tietê. *Boletim Paulista de Geografia*, **26**, 38–49.

Bastos E.G. de M., Araújo A.F.B. de, & Silva H.R. da (2005) Records of the rattlesnakes *Crotalus durissus terrificus* (Laurenti) (Serpentes, Viperidae) in the State of Rio de Janeiro, Brazil: a possible case of invasion facilitated by deforestation. *Revista Brasileira de Zoologia*, **22**, 812–815.

Buckup P.A. (2011) The eastern Brazilian Shield. *Historical biogeography of Neotropical freshwater fishes* (ed. by J.S. Albert and R.E. Reis), pp. 203–213. University of California Press, Ltd., Berkeley and Los Angeles.

de Barros L.C., Santos U., Cioffi M. de B., & Dergam J.A. (2015) Evolutionary Divergence Among *Oligosarcus* spp. (Ostariophysi, Characidae) from the São Francisco and Doce River Basins: *Oligosarcus solitarius* Menezes, 1987 Shows the Highest Rates of Chromosomal Evolution in the Neotropical Region. *Zebrafish*, **12**, 102–110.

Fitzpatrick S.W., Brasileiro C.A., Haddad C.F., & Zamudio K.R. (2009) Geographical variation in genetic structure of an Atlantic Coastal Forest frog reveals regional differences in habitat stability. *Molecular Ecology*, **18**, 2877–2896.

Gallego O.F. & Mesquita M. V. (2000) First record of oligocene conchostracans (Tremembé formation Taubaté Basin) from São Paulo, Brazil. *Journal of South American Earth Sciences*, **13**, 685–692.

Grazziotin F.G., Monzel M., Echeverrigaray S., & Bonatto S.L. (2006) Phylogeography of the Bothrops jararaca complex (Serpentes: Viperidae): past fragmentation and island colonization in the Brazilian Atlantic Forest. *Molecular Ecology*, **15**, 3969–3982.

Ingenito L.F.S. & Buckup P.A. (2007) The Serra da Mantiqueira, south-eastern Brazil, as a biogeographical barrier for fishes. *Journal of Biogeography*, **34**, 1173–1182.

Menezes N.A. (1970) Distribuição e origem da fauna de peixes de água doce das grandes bacias fluviais do Brasil. *Comissão Interestadual da Bacia Paraná-Uruguai. Poluição e piscicultura; notas sobre poluição, ictiologia e piscicultura* (ed. by I. de P. Secretaria da Agricultura), pp. 73–78. Faculdade de Saúde Pública da USP, São Paulo, SP.

Menezes N.A., Ribeiro A.C., Weitzman S., & Torres R.A. (2008) Biogeography of Glandulocaudinae (Teleostei: Characiformes: Characidae) revisited: Phylogenetic patterns, historical geology and genetic connectivity. *Zootaxa*, 33–48.

Ribeiro A.., Lima F.C.T., Riccomini C., & Menezes N.A. (2006) Fishes of the Atlantic Rainforest of Boracéia: testimonies of the Quaternary fault reactivation within a Neoproterozoic tectonic province in Southeastern Brazil. *Ichthyological Exploration of Freshwaters*, **7**, 157–164.

Ribeiro A.C. (2006) Tectonic history and the biogeography of the freshwater fishes from the coastal drainages of eastern Brazil an example of faunal evolution associated with a divergent continental margin. *Neotropical Ichthyology*, **4**, 225–246.

Serra J.P., Carvalho F.R. de, & Langeani F. (2007) Ichthyofauna of the rio Itatinga in the Parque das Neblinas, Bertioga, São Paulo State: composition and biogeography. *Biota Neotropica [online]*, **7**.

Vieira F. (2010) Distribuição, impactos ambientais e conservação da fauna de peixes da bacia do rio Doce. *MG.Biota: Boletim Técnico Científico da Diretoria de Biodiversidade do IEF – MG*, **2**, 5–22.

Vieira F., Santos G.B., Bernardo C., & Alves M. (2005) A ictiofauna do Parque Nacional da Serra do Cipó (Minas Gerais, Brasil) e áreas adjacentes. *Lundiana*, **6**, 77–87.

Vucetich M.G. & Ribeiro A.M. (2003) A new and primitive rodent from the Tremembé formation (late Oligocene) of Brazil, with comments on the morphology of the lower premolars of caviomorph rodents. *Revista Brasileira de Paleontologia*, **5**, 73–82.

Weitzman S.H., Menezes N.A., & Weitzman M.J. (1988) Phylogenetic biogeographyof the

Glandulocaudini (Teleostei: Characiformes, Characidae) with comments on the distributions of other freshwater fishes in eastern and southeastern Brazil. 379–427.
